# Realizing Minimally Perturbed, Nonlocal Chiral Metasurfaces for Direct Stokes Parameter Detection

**DOI:** 10.1021/acsnano.3c10749

**Published:** 2024-02-19

**Authors:** Yu Geun Ki, Byeong Je Jeon, Il Hoon Song, Seong Jun Kim, Sangtae Jeon, Soo Jin Kim

**Affiliations:** School of Electrical Engineering, Korea University, Seoul 02841, Republic of Korea

**Keywords:** Nonlocal resonance, Chiral metasurface, Stokes parameters, Mie resonance, Bound states in the continuum

## Abstract

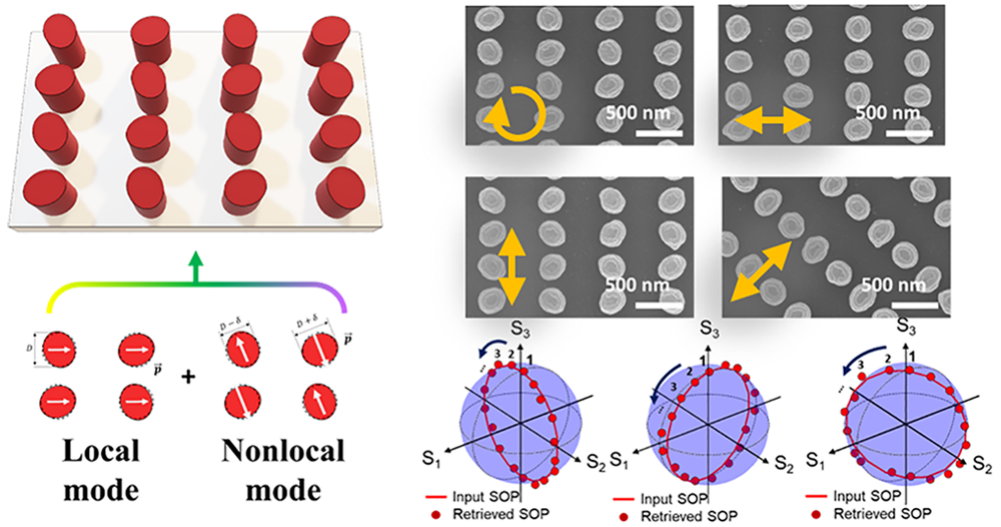

Recent development in nonlocal resonance based chiral metasurfaces draws great attention due to their abilities to strongly interact with circularly polarized light at a relatively narrow spectral bandwidth. However, there still remain challenges in realizing effective nonlocal chiral metasurfaces in optical frequency due to demanding fabrications such as 3D-multilayered or nanoscaled chiral geometry, which, in particular, limit their applications to polarimetric detection with high-Q spectra. Here, we study the underlying working principles and reveal the important role of the interaction between high-Q nonlocal resonance and low-Q localized Mie resonance in realizing effective nonlocal chiral metasurfaces. Based on the working principles, we demonstrate one of the simplest types of nonlocal chiral metasurfaces which directly detects a set of Stokes parameters without the numerical combination of transmitted values presented from typical Stokes metasurfaces. This is achieved by minimally altering the geometry and filling ratio of every constituent nanostructure in a unit cell, facilitating consistent-sized nanolithography for all samples experimentally at a targeted wavelength with relatively high-Q spectra. This work provides an alternative design rule to realizing effective polarimetric metasurfaces and the potential applications of nonlocal Stokes parameters detection.

A chiral metamaterial is an artificially fabricated, subwavelength structure designed by deliberately breaking geometric symmetry at the nanoscale. Due to the nature of structural chirality, it shows enhanced chiral light–matter interaction (LMI), also known as chiroptical response, which is typically characterized by several parameters such as circular dichroism (CD) and optical rotational dispersion (ORD). Contrary to naturally occurring chiral materials such as amino acids and deoxyribonucleic acid (DNA) which show extremely weak chiral LMI, chiral metamaterials have the capability to afford amplified and tunable chiroptical responses. In addition, their 2D counterparts, known as chiral metasurfaces, have the additional advantage of complementary metal-oxide-semiconductor (CMOS) compatible fabrication of their constituent elements which potentially enables practical chiral applications in diverse fields, including miniatured circular polarizers, biosensing with enhanced chiral field, CD spectroscopy, and quantum communications.^1−9^ Chiral metasurface plays a crucial role in the direct measurement of the Stokes parameters, which is the generalized method to evaluate the state of polarization (SOP) of light.^10−18^ To realize efficient chiral metasurfaces with strong CD, significant efforts have been made by exploiting various configurations of chiral geometries such as helix, twisted multilayer, slanted aperture, z-shape, and many other asymmetric structures.^19−27^ Despite the progress, there still remains the demand of effective design approaches of chiral metasurfaces by leveraging intriguing physical phenomena.

Meanwhile, in recent years, there has been growing interest in the development of nonlocal metasurfaces. Nonlocal metasurfaces have the ability to manipulate phase, wavefronts, and polarization of light in a relatively narrow spectral band by inducing collective oscillations of the constituent units over the supra-wavelength scale, in contrast to local metasurfaces which are operated by spatially localized resonances and have limited Q-factors.^28−38^ Following this, recently developed nonlocal chiral metasurfaces support the chiral resonance with strong CD and a high-Q factor, potentially offering a solution to realize chiral metamaterials with enhanced light–matter interaction at a narrow spectral range.^39−41^ Such nonlocal chiral metasurfaces provide potential applications to chiral emission, nonlinear optics, and optical communications with benefits over broadband ones in the fine-tuning of optical information within a limited bandwidth.^39,42,43^ One of the common and recent approaches to realizing nonlocal chiral metasurfaces is to induce quasi-bound states in the continuum (q-BIC) by designing the geometry to be broken mirror symmetry.^42−45^ Examples of these include the multi-perturbations in a double-layered metasurface^31,44^ and out-of-plane symmetry breaking using structures with different heights.^45−47^ More recently, nonlocal chiral metasurfaces in optical frequency were successfully demonstrated by designing and fabricating the in-plane chiral nanostructures at the subwavelength scale.^42,43^ Despite the progresses, the underlying physics and design rules of nonlocal chiral metasurfaces are not fully revealed compared to the case of local chiral metasurfaces explored for the last decades. In addition, it is still challenging to achieve high-Q stokes parameters detection since the precise and consistent-sized fabrication of the various nanogeometries for targeted S parameters and wavelength with high-Q factor is still elusive and thus has not been demonstrated.

Here, we address the challenges by designing one of the simplest types of nonlocal chiral metasurfaces with a high-Q factor by inducing nonlocal resonance and leveraging the optimal interaction with localized Mie resonance. We realize this by subtly perturbing elliptical nanoposts with geometric variations of 10% from the circular shape and elucidating the crucial role of Mie resonance^48,49^ supported by the aforementioned dielectric nanoposts. Furthermore, by leveraging the minimal variation of geometry that affords the near identical lithographic e-beam dose for all samples, we experimentally demonstrate the direct detection of each orthogonal point of the Poincaré sphere without complicated computational steps. Notably, contrary to typical Stokes metasurfaces consisting of various targeted geometries of nanostructures,^50,51^ we demonstrate that the subtle but precise perturbation^31,52^ of circular nanoposts affords the full coverage and direct tracking of the Poincaré sphere along the azimuth and ellipticity paths at a narrow spectral bandwidth with relatively high-Q resonances.

## Results and Discussion

**Figure 1:**
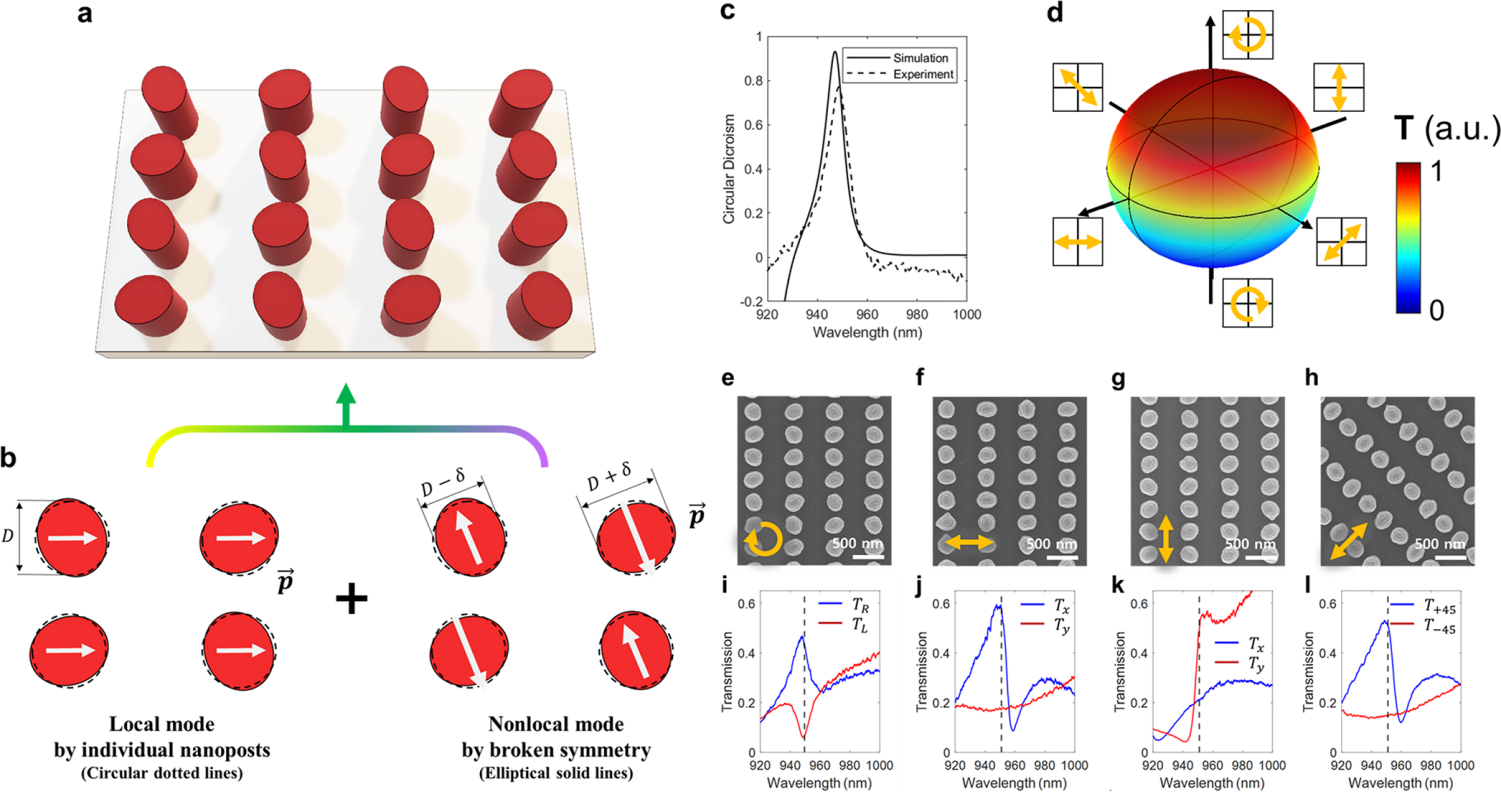
Proposed high-Q chiral metasurfaces realized by the subtle perturbation of circular nanoposts and their applications to nonlocal full Stokes polarization detection with minimal geometric variations. (a) Schematics of nonlocal chiral metasurface consisting of an array of elliptical nanopost positioned to enable simultaneous excitation of the local and nonlocal resonance modes. (b) Illustrations of the local (left) and nonlocal (right) modes involved to induce strong circular dichroism (CD). Nonlocal resonant mode is induced by the symmetry breaking with the perturbation δ from the diameter D and dipole orientation angle θ (outlined as solid lines). Notably, localized Mie resonance originally induced from the circular nanopost is negligibly altered by the deformation of the ellipses due to the geometric variation of 10% (outlined as a dotted line). (c) Simulated (solid line) and experimental (dotted line) values of CD for the RCP-targeted cell. (d) Corresponding transmission on Poincaré sphere for identifying S_3_ of Stokes parameters which track the ellipticity path. (e–h) SEM images of fabricated samples for Stokes parameters detection with minimal perturbations from circular nanoposts by the geometric variations less than 10% from circular nanoposts. (i–l)Measured transmission spectra for each targeted polarization and wavelength.

To demonstrate the suggested metasurface, we design and fabricate an array of nanoposts at a subwavelength scale using polysilicon (pSi) with a height of 330 nm (Figure 1). The detailed sizes of the designed nanostructures are described in Table 1. The designed metasurface exhibits a strong CD of 0.93 with a Q-factor over 100 at transmission spectra near 950 nm, as exhibited in Figure 1c, which is analyzed by the full-field simulations. The underlying working principle of the designed metasurface is schematically illustrated in Figure 1b. Starting with the design of a circular nanopost array which typically supports localized Mie resonances, we apply a subtle perturbation of the axis ratio to induce weakly coupled nonlocal resonances which induce oppositely oriented dipole arrays. The localized Mie resonance is negligibly altered by the perturbation, akin to the resonant condition of a circular nanopost (Supplementary Note 2). On the contrary, such weak perturbation induces high-Q resonance, which effectively interferes with Mie resonance to derive the optimal phase relationship necessary to realize efficient nonlocal chiral metasurfaces. We further reveal that the minimal geometric variations from circular nanoposts afford effective full Stokes parameters detection, as illustrated in Figure 1e–l. Figure 1e–h show the SEM images of fabricated samples of nonlocal metasurfaces which selectively respond to each targeted SOP, including the circular and linear polarizations. It is noteworthy that the filling ratio of constituent nanostructures at each unit cell is retained to be constant which affords the consistent-sized nanolithography with identical electron dose and enables reliable operation at a targeted wavelength and polarization with high-Q spectra for all samples.

**Table 1:** Dimensions of the Proposed Metasurfaces

Parameter	Dimension
D	240 nm
δ	25 nm
Height	330 nm
Period (x, y)	1250 nm, 700 nm
Refractive Index (Nanoposts)	3.59 + 0.001i
Refractive Index (Substrate)	1.45
Minimum Gap Between Adjacent Cell	108 nm
Filling Ratio	0.205
Aspect Ratio (Height/D)	1.37

**Figure 2:**
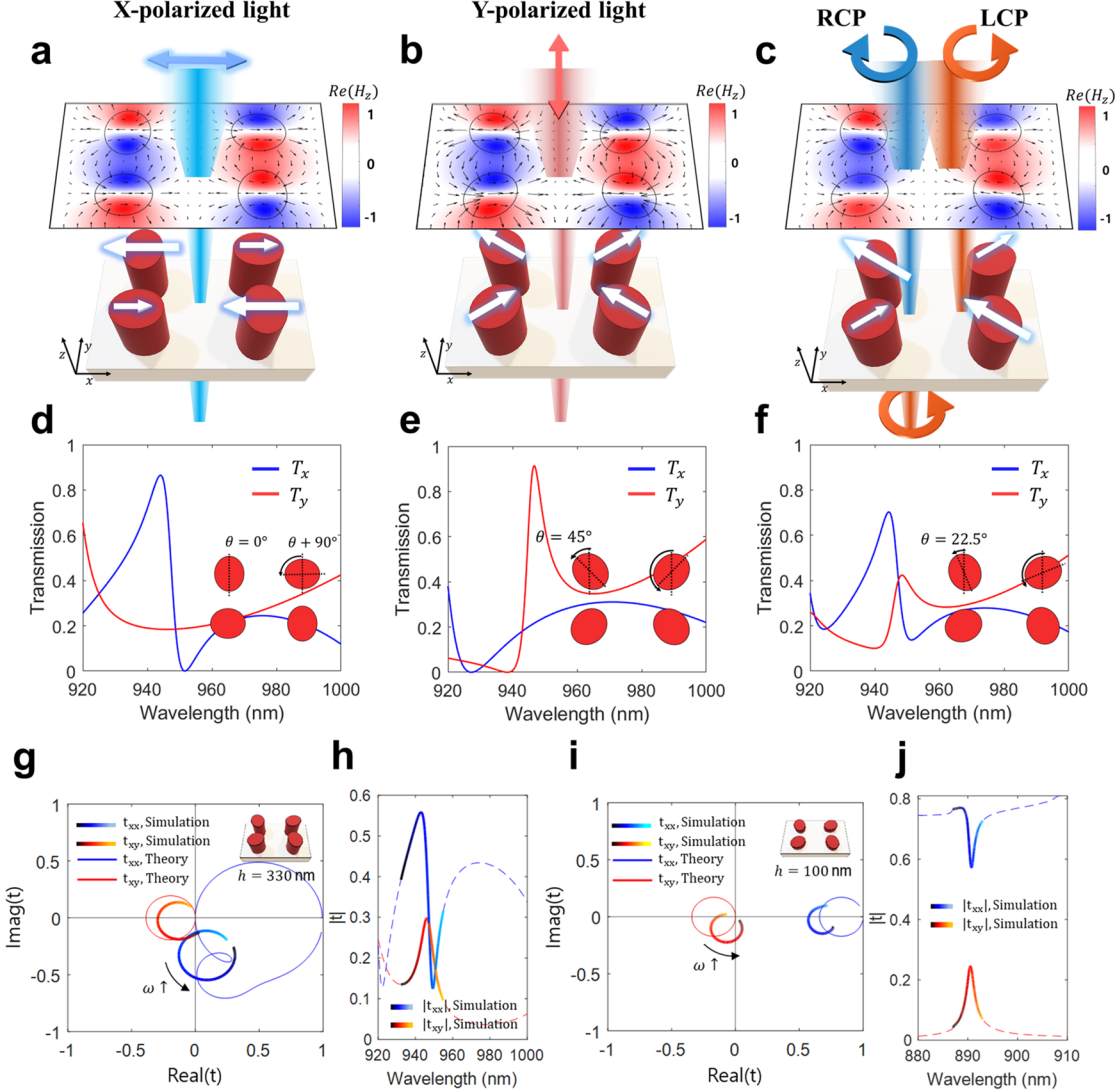
Analysis of the phase relationships of local and nonlocal modes induced from the designed chiral metasurface. (a–c) Three representative nanostructures and optical field images selectively resonating for the targeted polarizations of (a) *x*-, (b) *y*-directed, and (c) right-handed circular polarization at the wavelength of 947 nm. Oppositely directed electric dipoles are induced for each targeted polarization and support dark mode resonance as visualized by the magnetic fields oriented out of the plane by momentum transfer. (d–f) Simulated transmission spectra selectively displaying Fano resonance at targeted linear polarizations. The designed ellipses are depicted with oriented angles θ of 0°, 45°, and 22.5°, and adjacent ones are tilted by θ + 90°. (g, h) Complex amplitudes and spectra of the transmitted electric field of *x*-polarized light at the chiral metasurface. The phases of the *x*-directed field from copolarized (blue lines, Fano line-shape) and from cross-polarized transmission (red lines, Lorentzian line-shape) satisfy a 90° phase difference and result in either constructive or destructive interference by the additional 90° phase leading or lagging respectively under circular polarization. The gradient line colors depict the evolving transmission coefficients with changes in frequency. (i, j) Similar analysis of a chiral metasurface with the 100 nm-height of nanopost array, which is devoid of localized Mie resonance and does not satisfy the optimized phase relationships for strong CD.

To better understand the operating principles of the proposed metasurfaces, three representative nanostructures are modeled and analyzed to evaluate their coupling strength of resonance and mutual phase relationship. The first two types of metasurfaces are designed to selectively transmit two orthogonal, linearly polarized lights as illustrated in Figure 2a and b. By arranging the orientation of the long axis of ellipses to be the combination of 0° and 90° (Figure 2a), two oppositely *x*-directed resonant dipoles are induced and support dark mode resonance as visualized by the magnetic fields, which are oriented out of the plane by momentum transfer. Such resonance interacting with localized Mie resonance yields asymmetric Fano-shaped transmission spectra^53−55^ (Figure 2d). Additionally, the introduction of a rectangular lattice in the array induces birefringence, which precludes the resonance in *y*-polarization, as indicated by the red line, and enables high transmission contrast between the two orthogonally polarized lights (Supplementary Note 1). With a similar design approach, in Figure 2b, the metasurface is designed to selectively transmit *y*-directed linearly polarized light by applying axis orientations of 45° and 135° with comparable underlying optical phenomena (Figure 2e). For both cases, although the dark mode is selectively excited by each targeted polarization, the optical field profiles are close to identical where electric field circulates around the induced vertical magnetic dipoles by the similar nonlocal dark modes. The third type of metasurface is designed to exhibit strong CD, which is achieved by arranging the orientation of ellipses to be a combination of 22.5° and 112.5° (Figure 2c). This configuration induces the Fano resonance in both the *x*- and *y*-directed polarizations, as exhibited in Figure 2f, and the coherent constructive or destructive interaction between the induced resonances leads to the selective high-Q transmission of oppositely polarized circular states. Notably, despite the fact that the resonant mode profiles and geometric layout of the presented metasurfaces show close similarity, supporting the nonlocal dark mode, there is a stark difference in the interaction with the targeted linear or circular polarization.

To determine the resonant phase relationship of the demonstrated chiral response, the transmission coefficient is analyzed and displayed on the complex plane as illustrated in Figure 2g.^56^ We start by examining the phases and amplitudes of copolarized and cross-polarized components from the transmitted, *x*-polarized light. The tiny circle in the fourth quadrant characterized by Fano resonance overlaps the large circle introduced by near critically coupled Mie resonance as indicated by the blue line, which is the copolarized transmission and expected resonant curve based on coupled mode theory. In addition, due to the subtle mirror symmetry breaking of our proposed metasurface (Figure 2c), *x*-directed cross-polarized light is induced during the occurrence of weakly coupled nonlocal Fano resonance from *y*-polarized light incidence, as indicated by the red lines in Figure 2g. It is noteworthy that the identical phenomenon can be created in the array of ellipses with C_2_ and mirror symmetry breaking (Supplementary Note 4). At the operating wavelength of interest, the phases of *x*-directed copolarization (T_*xx*_) and cross-polarization (T_*xy*_) approximately satisfy a 90° difference, and the additional 90° phase leading or lagging under circular polarization results in either constructive or destructive interference between the cross- and copolarized components of circularly polarized light, which ultimately leads to the strong and high-Q CD. Such phase condition for coherent interaction is validated in the designed nonlocal chiral metasurfaces using full-field simulation at the targeted spectrum of Fano resonance as displayed by solid lines in Figure 2d–f. Importantly, localized Mie resonance plays an essential role in realizing effective high-Q chiral metasurfaces. As a counter-comparison, a chiral metasurface devoid of Mie resonance is evaluated by reducing the height of the nanopost to 100 nm. The evaluation of the transmission coefficient reveals that the phase relationship between co- and cross-polarizations by high-Q nonlocal resonance does not satisfy the ideal criterion (Figure 2i,j) and exhibits a significant decrease of CD as the height decreases further (Supplementary Note 3). The slight deviations between the simulated and theorical analyses stem from the nonideal factors such as overlap of Mie resonances and rotation of transmission coefficients on a complex plane induced by the out-of-plane symmetry breaking due to the existence of SiO_2_ substrate.^57^

**Figure 3:**
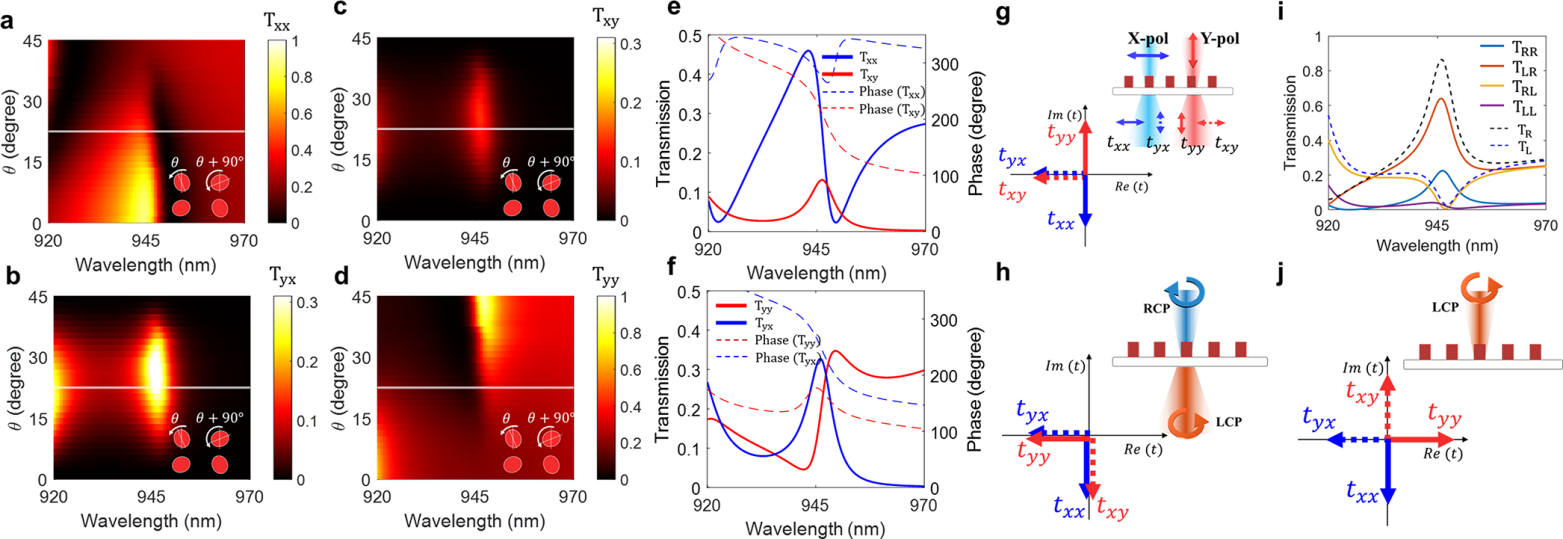
Optimization of chiral metasurfaces with vectorial analysis of complex transmission coefficients. (a, b) Transmission spectra for continuously changing orientation angles (θ) of ellipses under *x*-polarized incident light. (a) X-directed, copolarized transmission shows a Fano line-shape, and (b) *y*-directed, cross-polarized transmission shows a Lorentzian line-shape within the overall range of orientation angles. (c, d) Transmission spectra under *y*-polarized incident light with (c) *x*-directed cross-polarization and (d) *y*-directed copolarization. The angle of 22.5° (white line) satisfies the optimized conversion to cross-polarization to maximize CD. (e, f) Transmission amplitudes (solid lines) and their respective phases (dotted lines) at the angle (θ) of 22.5°. (e) Transmission components with *x*-directed polarization (T_*xx*_ and T_*xy*_) satisfy the matching of amplitudes and 90° phase difference. (f) Transmission components with *y*-directed polarizations (T_*yx*_ and T_*yy*_) satisfy similar optimized conditions. (g) Schematical mapping of the transmission components of linearly polarized incidence on a complex plane (λ = 947 nm). Importantly, copolarized components (T_*xx*_ and T_*yy*_, blue and red solid lines, respectively) satisfy 180° phase difference by the birefringence effect induced by the rectangular lattice, and corresponding cross-polarized light (T_*yx*_ and T_*xy*_, blue and red dashed lines, respectively) shows the phase leading or lagging of 90° with respect to the copolarized components. (h) RCP incidence with additional phase lagging of 90° gives the in-phase relationship. (j) LCP incidence with phase leading of 90° gives the out-of-phase relationship. (i) Simulated transmission components of the designed metasurface.

As the next stage, we maximize the generation of cross-polarization and optimize the phase relationship by sweeping the orientation angle (θ) of elliptical nanoposts (Figure 3). As illustrated in Figure 3a–d, the transmission spectra of co- (T_*xx*_ and T_*yy*_) and cross-polarized light (T_*yx*_ and T_*xy*_) are calculated under *x*- and *y*-polarized incident light. Enhanced transmissions of copolarization with the Fano line feature are observed as the orientation angle (θ) changes toward 0° for *x*-polarized incident light (Figure 3a) and toward 45° for *y*-polarized incident light (Figure 3d), respectively. At an angle of 22.5°, maximized cross-polarized transmission is induced (Figure 3b,c), while the Fano spectra of copolarization still remain. Figure 3e shows the cross-sectional spectra, i.e., the spectra along the white lines in the transmission panels, at the optimized angle of 22.5°. It is observed from the figure that the transmission of Fano resonance (blue solid line) shows the under-coupled condition with a phase variation of less than 180° (blue dotted line). On the contrary, the transmission of cross-polarized light features a Lorentzian curve, and the corresponding phase evolves from 0° to 180°, which leads to the optimized phase relationship of a 90° difference between co- and cross-polarization at the operating wavelength of 947 nm. The optimum phase relationship is likewise satisfied by transmission under *y*-polarized incident light (Figure 3f).

To additionally emphasize the role of birefringence at localized Mie resonance (Supplementary Note 1), we schematically plot the transmission vector of each component of polarization (Figure 3g–j). As aforementioned, *x*-directed copolarization (solid blue line, T_*xx*_) induces *y*-directed cross-polarization (dotted blue line, T_*yx*_) with a 90° phase delay, and similarly, *y*-directed copolarization (solid red line, T_*yy*_) causes *x*-directed cross-polarization (dashed red line, T_*xy*_). Crucially, the copolarized components (T_*xx*_ and T_*yy*_) satisfy the 180° phase difference approximately by the birefringence effect induced by the rectangular lattice (Figure 3g), and under RCP incidence, additional phase lagging of 90° leads to the in-phase relationship (Figure 3h), whereas under LCP incidence, the phase leading of 90° gives the out-of-phase relationship (Figure 3j) for all components. Each polarization component of transmitted light is exhibited in Figure 3i. The nonzero transmission of T_RR_ stems from nonideal conditions of phase and amplitude relationships from the resonant modes of dielectric nanostructures.

**Figure 4:**
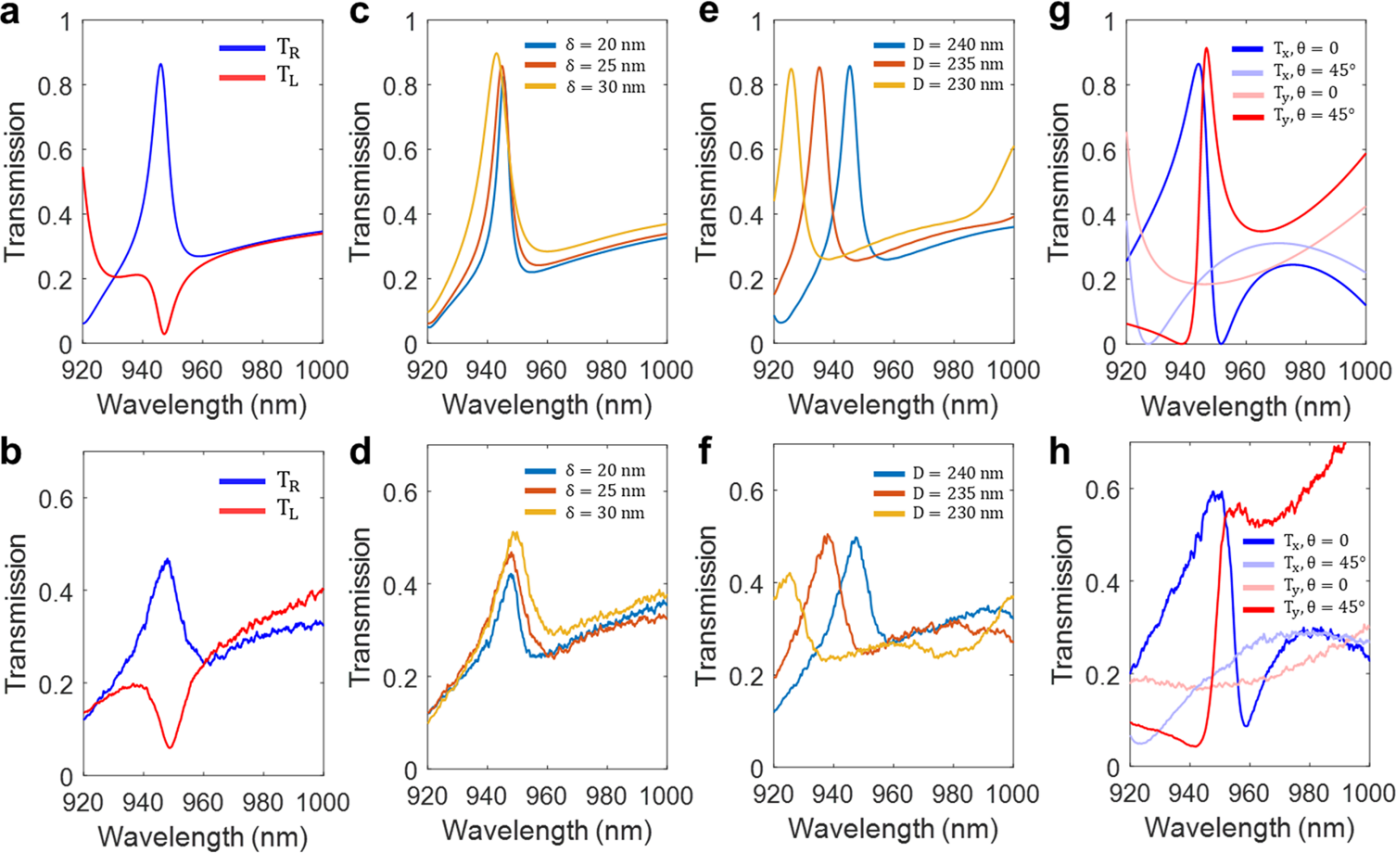
Simulated and experimental transmission of the nonlocal Stokes metasurface. (a, b) Transmission spectra of nonlocal chiral metasurface (θ = 22.5°) under the incident light with RCP (blue line) and LCP (red line). (c, d) Transmission spectra with the increased perturbation of aspect ratio (δ) for the control of Q-factor. (e, f) Transmission spectra with different diameters (D) for the control of the operating wavelengths. (g, h) Transmission spectra of the metasurfaces with different axis orientations (θ = 0° or 45°) for the detection of *x*- (red) or *y*-polarized light (blue). (a, c, e, g) The first row is the simulated analyses, and (b, d, f, h) the second row is the experimental measurements.

To experimentally verify the design rule, we measure the transmission spectra of the fabricated chiral metasurface samples. We fabricate a set of chiral metasurfaces by tuning the design parameters to control and detect targeted optical properties. We first measure the transmission of a representative chiral metasurface under oppositely polarized circular waves. As exhibited in Figure 4a and b of simulated and experimental spectra, they show the enhanced and suppressed transmission for targeted polarizations with a Q-factor experimentally determined to be 77.78. A relatively lower Q-factor than the simulated expectation stems from the surface roughness and reduced collective resonances caused by the limited sample size. Subsequently, we investigate the effect of the axis ratio and size of the constituent elliptical nanopost on tuning the Q-factor and operating wavelength of the nonlocal resonance, respectively. By adjusting the perturbation, i.e., the axis difference (δ)^31,52^ to be 20, 25, and 30 nm, the Q-factor of transmitted light is changed from 65 to 105 keeping the operating wavelength constant (Figure 4c,d). The tiny changes of resonant peak’s spectral positions are related to the fabrication imperfections and small variations of nanopost volumes as δ varies (Supplementary Note 15). Following that, the size of the ellipse is changed while the remaining parameters, such as orientation angle (θ) and axis difference (δ), are held constant, which results in the shifting of the operating wavelengths to the targeted spectral region (Figure 4e,f). Finally, to tune the response to the polarizations, we change the orientation angles (θ) of the constituent ellipse to 0° and 90° or 45° and 135° to detect *x*- or *y*-directed linear polarizations, respectively. As shown in Figure 4g and h, enhanced transmission with a Fano line-shape is observed in each targeted polarization. The opposite symmetric factor in Fano line-shape^47−49^ between the two metasurfaces is due to the 180° phase difference of Mie resonance caused by the birefringence effect. The measured transmission contrast and CD can be enhanced further by improving the quality of fabricated samples including surface roughness and size variations between the individual nanoposts (Supplementary Note 12).

**Figure 5:**
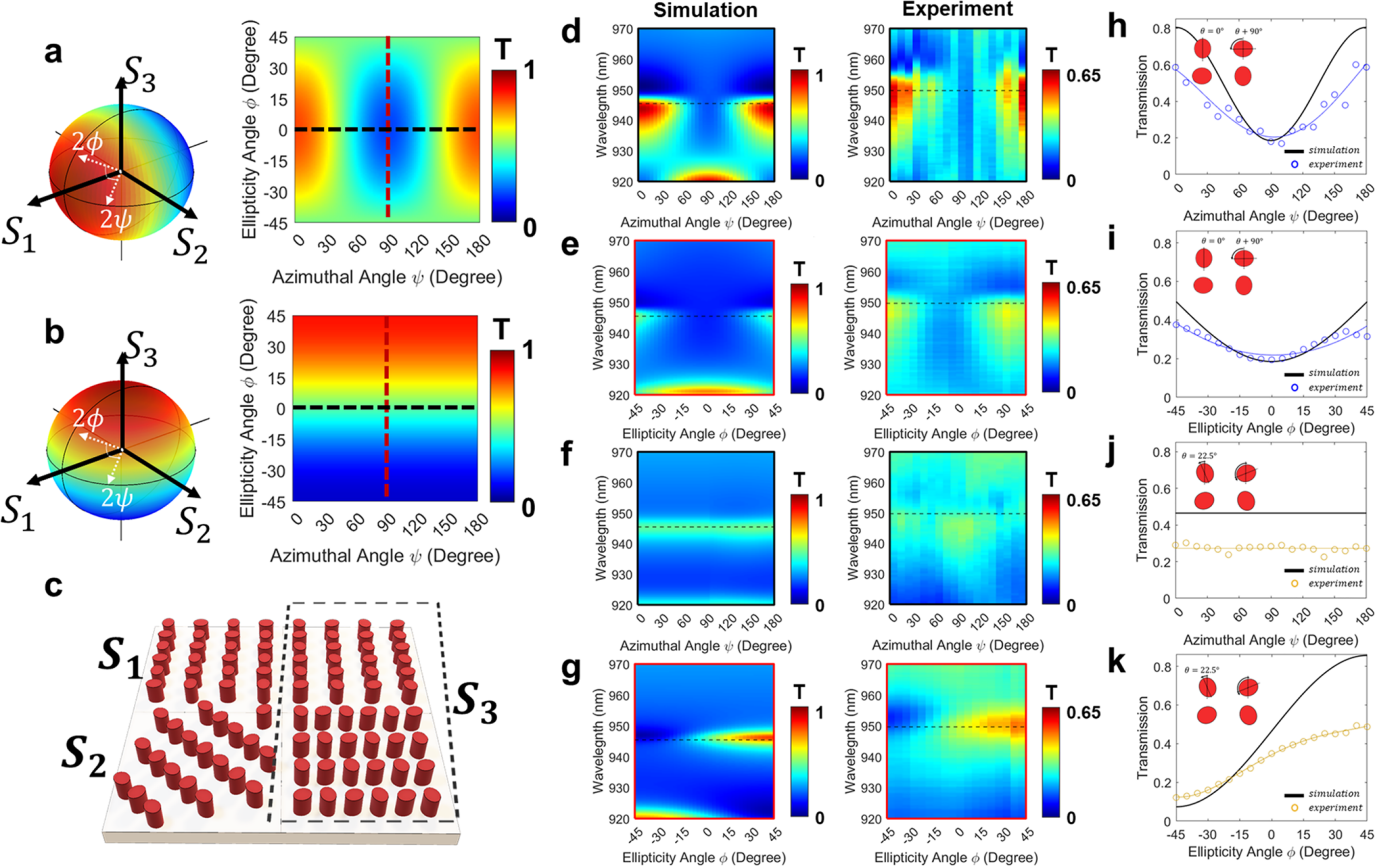
Measurement of the states of various polarizations along the directions of azimuthal and ellipticity paths of Poincaré spheres. (a) Simulated transmission of the metasurface (orientation angle θ of 0°) at the Poincaré spheres with targeted wavelength of 946 nm for the tracking of azimuthal angles. (b) Simulated transmission of the metasurface (orientation angle θ of 22.5°) for the tracking of ellipticity angles. (c) Schematic illustration of the nonlocal metasurfaces for measurement of the states of various polarizations. (d, e) Profiles of the simulated (left section) and experimental (right section) transmission spectra obtained by monitoring along the (d) azimuthal and (e) ellipticity paths of Poincaré sphere at metasurface for azimuthal angle detection, i.e., along the vertical and horizontal dotted line at the panel of d and e, respectively. (f, g) Profiles of the simulated (left section) and experimental (right section) transmission spectra obtained by monitoring along the (f) azimuthal and (g) ellipticity paths of Poincaré sphere at metasurface for ellipticity angle detection, i.e., along the vertical and horizontal dotted line at the panel of a and b, respectively. (h–k) Transmission values at the targeted wavelength (cross sectional lines of d, e and f, g) obtained by monitoring along the azimuthal and ellipticity angles at each metasurface for (h, i) azimuthal and (j, k) ellipticity angle detection, respectively. Solid lines indicate simulated analyses, and circles with guided line indicates experimental measurements.

Using the combination of our proposed metasurfaces, we demonstrate the measurements of the states of various polarizations along the directions of azimuthal and ellipticity angles, as exhibited in Figure 5. Notably, due to the subtly perturbed geometries of nanostructures, each parameter to tune the optical properties is minimally correlated and can be controlled in a relatively independent fashion. As seen in Figure 5a and b, by optimally orienting the axis angles while keeping the lattice size and nanostructure’s filling ratio fixed, we realize the effective detection of each orthogonal corner of the Poincaré sphere at the targeted narrowband spectrum. For example, the metasurface with an orientation angle (θ) of 0° directly tracks the azimuthal path, which maximizes *x*-polarized light transmission and minimizes *y*-polarized light transmission (Figure 5a). The metasurface with an orientation angle (θ) of 45° also can be employed for azimuthal angle, but it maximizes *y*-polarized light transmission. With a similar approach, the chiral metasurface with an orientation angle (θ) of 22.5° tracks the ellipticity path, which maximizes and minimizes the RCP and LCP transmission, respectively (Figure 5b). Note that the combination of the two orthogonal forms of the chiral metasurfaces eliminates the fluctuations of transmission along the azimuthal path near the equator to be zero (Supplementary Note 9). Full Stokes parameters detection for fully polarized light can be achieved by integrating the azimuthal and ellipticity angles acquired through this way with the total intensity of the light derived from the SiO_2_ substrate without any patterns.^9,12^

To experimentally verify the abilities of polarimetric detection with a relatively high-Q factor, transmission spectra at the fabricated metasurfaces for various polarization states are measured and plotted as profile maps. Figure 5d–g illustrate the sets of spectra for various angles along the azimuthal and ellipticity paths of Poincaré sphere. Figure 5d and e show the simulated and experimental spectra of the metasurface with an orientation angle (θ) of 0° under the variations of incident polarization along the azimuthal (Figure 5d) and ellipticity (Figure 5e) angles. Both experiments and simulations indicate that the Fano-shaped spectra show the maximum transmission value at 0° and 180° along the azimuthal angles tracking with a full width at half maximum (FWHM) of 13.5 nm in simulation and 17 nm in the experiment. Simulated and experimental results for the variations along the ellipticity path are also presented and show close similarity between the simulated analyses and experimental measurements. Figure 5f and g illustrate the spectra of the metasurface with an orientation angle (θ) of 22.5°. The transmission exhibits the highest value at an ellipticity angle of 45° which corresponds to RCP and gradually decreases as it approaches LCP. On the other hand, the transmission spectra along the azimuthal path show the constant values with minimal variations in experimental measurements. Experimental results demonstrate trends consistent with the simulated analyses. The FWHM at the target SOP (ellipticity angle = 45°) is 6.7 nm in the simulation and 12.2 nm in the experiment. Figure 5h and j show the cross-sectional normalized transmission spectra along the horizontal dotted lines in the profiles of Figure 5a and b, respectively, which show the consistent trend of variations along the azimuthal angles and close similarity between the simulated analyses (black solid line) and experimental measurements (empty circles). And, Figure 5i and k show the cross-sectional normalized transmission spectra along the vertical dotted lines, in which the overall transmission of the metasurface for ellipticity angle detection monotonically increases as the ellipticity angle increases (Figure 5k). Relatively lower transmissions in the experiments are due to the nonideal factors including fabrication imperfection and loss of light by surface scattering.

**Figure 6:**
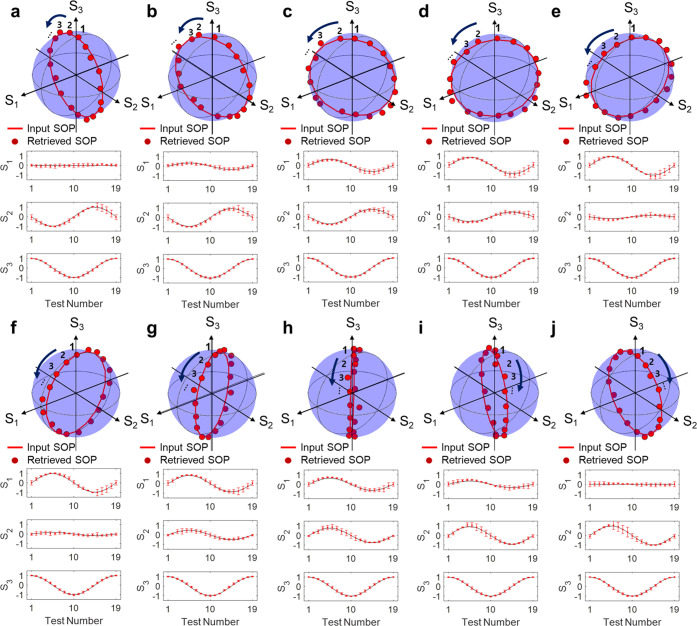
Extracted SOPs from fabricated nonlocal metasurfaces. (a–j) Retrieved SOPs from the fabricated metasurfaces under arbitrarily complex polarizations. Stokes parameters are extracted from the intersecting points on the Poincaré sphere from the constituent metasurfaces with a numerical analysis provided in Supplementary Note 10. The solid red lines represent the polarization inputs, and the red dots represent measurements values. Four identical measurements are conducted to evaluate the mean values with standard deviations at each test set.

Finally, arbitrarily polarized states of light including linear and circular polarizations are measured by tracking intersecting points at the Poincaré sphere from the transmission of the constituent metasurfaces. Calculation of Stokes parameters is numerically expressed by the Eq. S2 and detailed retrieval method in Supplementary Note 10. Figure 6 displays the retrieved SOPs from the fabricated metasurfaces under various complex polarizations. The solid red lines on the Poincaré sphere represent the polarization input, and the corresponding measurement results are depicted as red dots. We performed measurements for all points on the Poincaré sphere at the targeted wavelength of 950 nm with the intervals of azimuthal and ellipticity angles to be 10 degrees, respectively. All experimental results closely match the actual SOPs with the slight differences of 0.047, 0.049, and 0.024 on average at the metasurfaces for S_1_, S_2_, and S_3_, respectively. Further analysis of the statistical errors including standard deviations is analyzed in Supplementary Note 11.

## Conclusion

To summarize, we present nonlocal chiral metasurfaces by subtly perturbing the circular geometry and leveraging the optimized interaction between nonlocal and local resonances. With these, we demonstrate effective polarimetric detection with the direct tracking of azimuthal and ellipticity paths in the Poincaré sphere. Such effective detection with a relatively high-Q factor can be realized by minimally altering the geometries of constituent nanostructures while keeping their filling ratio in a unit cell constant, which affords consistent-sized nanofabrication and facilitates effective operation at a targeted wavelength and polarization experimentally for all samples. We believe the presented study and approach can be a solution to realize practical nonlocal chiral metasurfaces with potential applications to molecular sensing, polarimetric imaging, nonlinear optics, and optical communication.

## Method

### Optical Simulation

To evaluate the optical properties, simulated analysis using the finite-difference time-domain (FDTD) method is performed. Arrays of silicon elliptical nanopost with 330 nm height (refractive index of 3.59 + 0.001i) are arranged on the SiO_2_ substrate (refractive index of 1.45), and the period of arrays is designed as 1250 and 700 nm for *x*- and *y*-directions, respectively. Elliptical geometry is designed with the sizes of the long and short axis to be 265 and 215 nm, respectively. Orientation angles of the long axis are the combination of 22.5° and 112.5° for the chiral metasurface, 0° and 90° for the metasurface with the transmission of *x*-directed polarization, and 45° and 135° for the metasurface with the transmission of *y*-directed polarization. In the unit cell, the periodic boundary condition is applied in the *x*- and *y*-direction, and the perfectly matched layer absorbing boundary conditions are applied along the *z*-direction. Transmission is calculated by monitoring transmitted power in a far-field regime.

### Device Fabrication and Measurements

We fabricated the array of elliptical nanostructures operating at the near-infrared wavelength. The 330 nm thick poly-Si was deposited by LPCVD (low-pressure chemical vapor deposition) process on the quartz substrate followed by electron-beam lithography (EBL) to define the elliptical shape. Next, a 30 nm thick Cr hard mask was deposited by e-beam evaporation, followed by a lift-off process. The remaining elliptical Cr pattern was used as a mask. Finally, poly-Si was etched with an RIE (reactive ion etching) machine and the remaining Cr pattern was removed by wet-etching. All the measurements were done by frontside illumination from air to the quartz substrate. Transmission spectra of fabricated devices were measured with a confocal optical setup coupled to collimation lensed fiber and spectrometer. The illumination of light was made by using an unfiltered supercontinuum source (NKT Photonics, SuperK EVO). To detect all Stokes parameters, input SOPs of each experimental set was generated by rotating linear polarizer by 10° ranging from 0° to 180° while keeping the angle of quarter waveplate fixed. Such experimental set was conducted for the total 10 different angles of quarter waveplate ranging from −45° to 45° with 10° intervals to fully cover Poincaré sphere. The measurements are repeated 4 times under identical sets of input SOPs to obtain a statistically averaged data set.

## Supporting Information Available

Analysis of Mie resonances, control of Q factor, lattice periodicity, pure nonlocal resonance, multipole decomposition, C_2_ group cells, detection of S_3_ parameter, direct Stokes parameters measurements, statistical methods for Stokes parameters retrieval, impact of fabrication imperfections, fabrication steps, impact of unit cell number, effect of overcut shape with various degrees of perturbations, experiments for Jones matrix transmission spectra, experimental setup, dependence on incident angle of metasurfaces, comparison with previously reported chiral metasurfaces, nonlocal chiral metasurfaces for oblique excitation, dependence of circular dichroism on extinction coefficient by absorption (PDF)
