# Supplementary material for: Realizing Minimally Perturbed, Nonlocal Chiral Metasurfaces for Direct Stokes Parameter Detection
Source: ACS Nano. 2024 Feb 19;18(9):7064–73. doi: 10.1021/acsnano.3c10749 (PMC10919284; doi:10.1021/acsnano.3c10749)
Supplement: Supplementary file 1 — nn3c10749_si_001.pdf [file nn3c10749_si_001.pdf]

Supporting Information

Realizing Minimally Perturbed, Nonlocal Chiral Metasurfaces for Direct Stokes Parameter Detection

*Yu Geun Ki, Byeong Je Jeon, Il Hoon Song, Seong Jun Kim, Sangtae Jeon, and Soo Jin Kim**

School of Electrical Engineering, Korea University, Seoul 02841, Republic of Korea

Email: kimsjku@korea.ac.kr

Supplementary Note 1. Analysis of the localized Mie resonances and birefringence effects

Localized Mie resonances are supported in the designed metasurface by constituent silicon nanostructures. The designed size of a silicon nanopost is 240 nm in diameter (D) and 330 nm in height (h) as depicted in the inset of **Figure S1a**, which supports both an electric dipole (ED) and magnetic dipole (MD) resonances. To induce a chiral response, it is necessary to create a phase difference of 180° between two orthogonal, linearly polarized lights. To achieve this, we analyze the phase and amplitudes of transmitted light for various periods (P) between the nanostructures, as visualized in the transmission profile of **Figure S1**.

In **Figure S1a**, it is observed that both the ED and MD resonances with critically coupled conditions are supported at the wavelengths of 900 nm and 1040 nm for the period (P) of 625 nm. As the period decreases, the MD resonance mode is relatively more blue-shifted than the ED resonance mode, and at the period of P = 350 nm, the two modes overlap and reach the status of the over-coupling condition. To induce a birefringence effect, we design a rectangular lattice by determining the periods Px and Py to be 625 nm and 350 nm, respectively. **Figure S1b** presents the amplitude and phase of transmitted light results. The blue line represents the transmittance (solid line) and phase (dotted line) for x-polarized light, while the red line represents the results for y-polarized light. For x-polarized light (blue line), abrupt phase changes are observed at the two frequencies of critically coupled resonant modes, and, for y-polarized light (red line), the overlap of the two resonant modes causes a gradual change of phase within the indicated wavelength range of interest. Such phase differences result in an overall phase difference of 180°

between the two orthogonally polarized lights and a birefringence effect at the shaded spectra region.

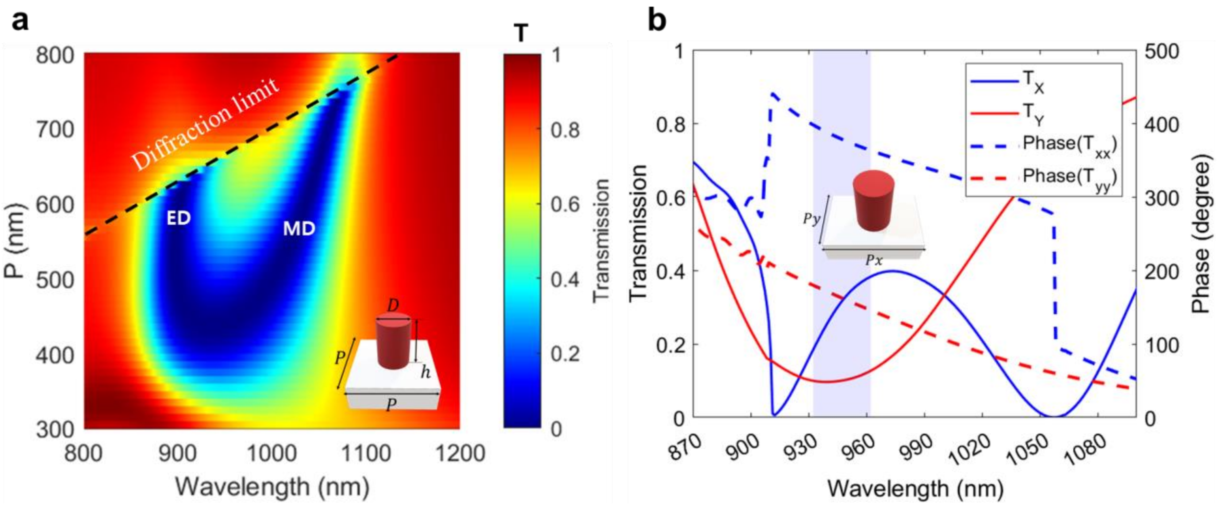

Figure S1. Analysis of the localized Mie resonances **a.** Transmission spectra of metasurfaces for the continuously varying periods (P) of a square lattice. **b.** Transmission and the corresponding phase profiles under the incident light with x- and y-directed, linear polarizations. The shaded region indicates the spectral band of interest with positive circular dichroism (CD) at the designed chiral metasurface.

Supplementary Note 2. Analysis of the impact of geometric perturbations on the localized Mie resonances.

To analyze the impact of geometric perturbations on the localized Mie resonances, transmission amplitude and phase under x- and y-directed polarizations are analyzed. As illustrated in **Figure S2a** and **b**, the degree of perturbation denoted by the δ value is continuously changed from zero to 30 nm. As the perturbation increases, the excitation of sharp spectral features stemming from the nonlocal mode is observed at the wavelength of 950 nm, which is overlaid on the broadband spectral features of localized Mie resonances. It is noteworthy that the birefringence effect induced by the rectangular lattice is negligibly altered by the perturbation and remains largely unchanged with a relative phase difference of 180° (**Figure S2c**). Additional excitation of weakly coupled nonlocal resonance induces a sharp change of phases due to an under-coupled condition at the operating wavelength of 950 nm. Except for the spectral point of such an under-coupled condition, the birefringence caused by the localized resonance is maintained akin to the unperturbed metasurface and satisfies the condition of an optimized phase relationship.

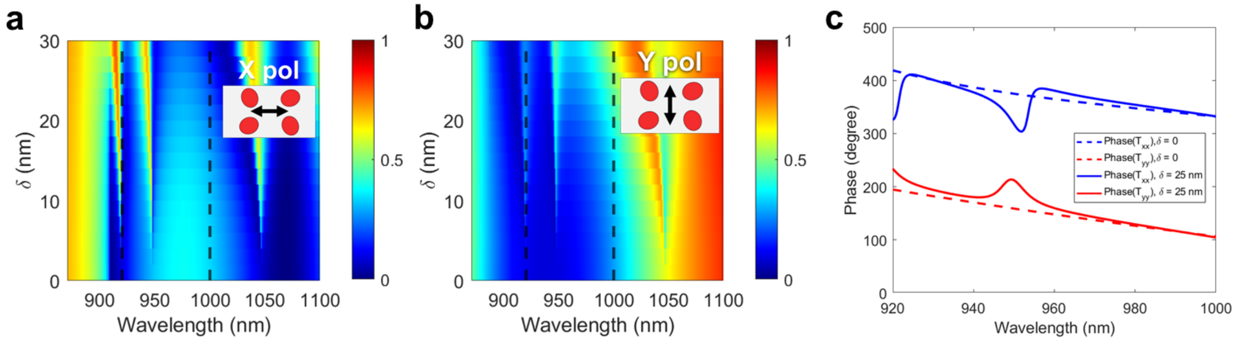

Figure S2 Analysis of the localized Mie resonance under the subtle perturbation of an axis ratio **a, b.** Transmission spectra of metasurface for the continuously varying perturbation of the axis ratio (δ) under x- (a) and y-directed (b) linear polarizations. **c.** the phase profiles of the unperturbed metasurface (dotted) and the perturbed metasurface with δ = 25 nm (solid) under the incident light with x- and y-directed linear polarizations. The spectral range of **c** is indicated as the dotted line at the colormap (a) and (b).

Supplementary Note 3. Role of Mie resonances for strong CD

The localized Mie resonance plays an important role in enhancing strong CD. Firstly, it generates a reflection band, and combining it with nonlocal mode enables the generation of a transmission peak. Secondly, it induces a phase difference of about 90° between co-polarized and cross-polarized light, enabling optimized CD in transmission.

To investigate the presence or absence of localized Mie resonance, we conduct an analysis by varying the height of the nanopost while keeping other geometric parameters constant as illustrated in **Figure S3**. It is observed that the nonlocal mode caused by the broken geometric symmetry remains regardless of the variations in height. However, the CD signal is significantly affected and altered depending on the values of height as indicated in **Figure S3c** and the representative regions highlighted by dotted circles.

At a height larger than 150 nm, localized Mie resonance is supported and a strong CD signal is observed by the difference in transmission between two oppositely polarized circular waves. On the contrary, at the height of 100 nm, although there is no background reflection supported by local resonance, the nonlocal mode (bottom circle) still exists. However, circular dichroism (CD) is not observed in this condition. This indicates that CD starts to emerge when the height increases and crosses a specific threshold value to induce a local resonance. This analysis implies that the optimal interaction between a nonlocal resonance and a localized Mie resonance plays a crucial role in the optimized condition of a strong CD.

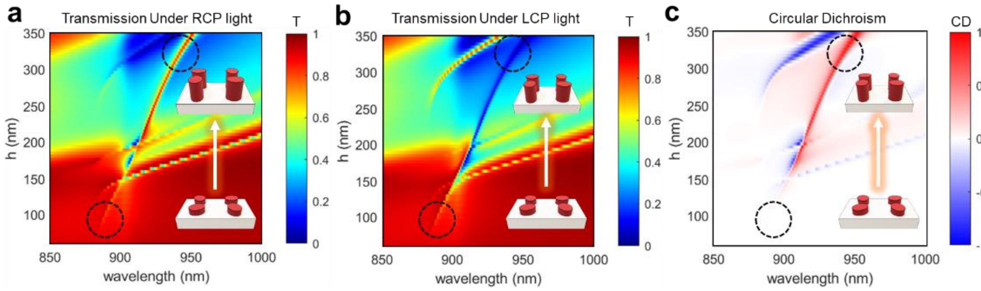

Figure S3. Role of Mie resonances for strong CD **a, b.** Transmission spectra for various heights of silicon nanopost under the incident light with the polarizations of RCP (**a**) and LCP (**b**), respectively. The geometry with C_4 symmetry breaking induces nonlocal mode irrespective of the variations of height. **c.** CD spectra of corresponding metasurfaces. As observed in the two regions indicated as dotted lines, CD does not arise if the local mode is not supported by the nanostructure with a relatively lower height approximately less than 150 nm, and a strong CD signal appears above the threshold height condition.

Supplementary Note 4. Control of Q-factors with the variations of δ

The nonlocal modes caused by the symmetry breaking exhibit different coupling strengths to light depending on the degree of perturbation. As the greater perturbation applies, light is more strongly coupled which is quantified by Q-factors. To analyze this aspect, we present the changes in transmittance with respect to the degree of symmetry breaking as illustrated in **Figure S4a** and **b**. As mentioned in the main text, we achieve symmetry breaking by reducing the size by a subtle value (δ) to form a minor axis and increasing the diameter by the same value to form a major axis. The colormap in **Figure S3a** and **b** illustrate the variations in transmittance spectra for the various changes in δ values. It is evident that the region of strong transmission under right-handed circular polarization (RCP) (a red highlighted region in **Figure S4a**) and the region suppressed under left-handed circular polarization (LCP) (deep blue region in **Figure S4b**) features a gradual increase of FWHM in transmissions as δ increases.

The asymmetry parameter, which represents the degree of symmetry breaking, exhibits an inverse quadratic relationship with the Q-factors[1]. In our design, we define the asymmetry parameter α as follows.

$$\alpha = \frac{2\delta}{D}$$

Based on this, we calculate Q-factors for each metasurface as displayed in **Figure S4c**, which shows the consistent matching between the simulated and theoretical values.

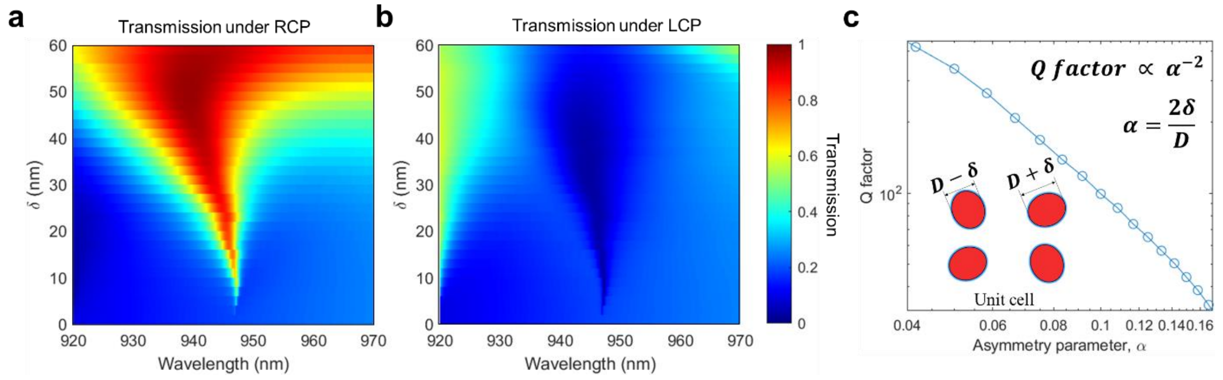

Figure S4. Transmission spectra for various δ values **a.** transmission spectra under RCP polarized incidence. **b.** under LCP polarized incidence, and **c.** simulated Q-factors for the structures of different asymmetry parameters.

Supplementary Note 5. Analysis of the lattice periodicity

To find the effect of lattice size on the nonlocal property, **Figure S5** illustrates the transmission contrast and CD for various periodicities of nonlocal chiral metasurfaces. Overall, as the period increases, relatively sharp transmission peaks under RCP are continuously red shifted. There is a more predominant shift of the resonant peak by the increase of the y-directed period than the increase of the x-directed periods. This implies that the nonlocal resonance is mainly induced by the interaction with y-directed adjacent nanopost and is thus more sensitive in the period Py.

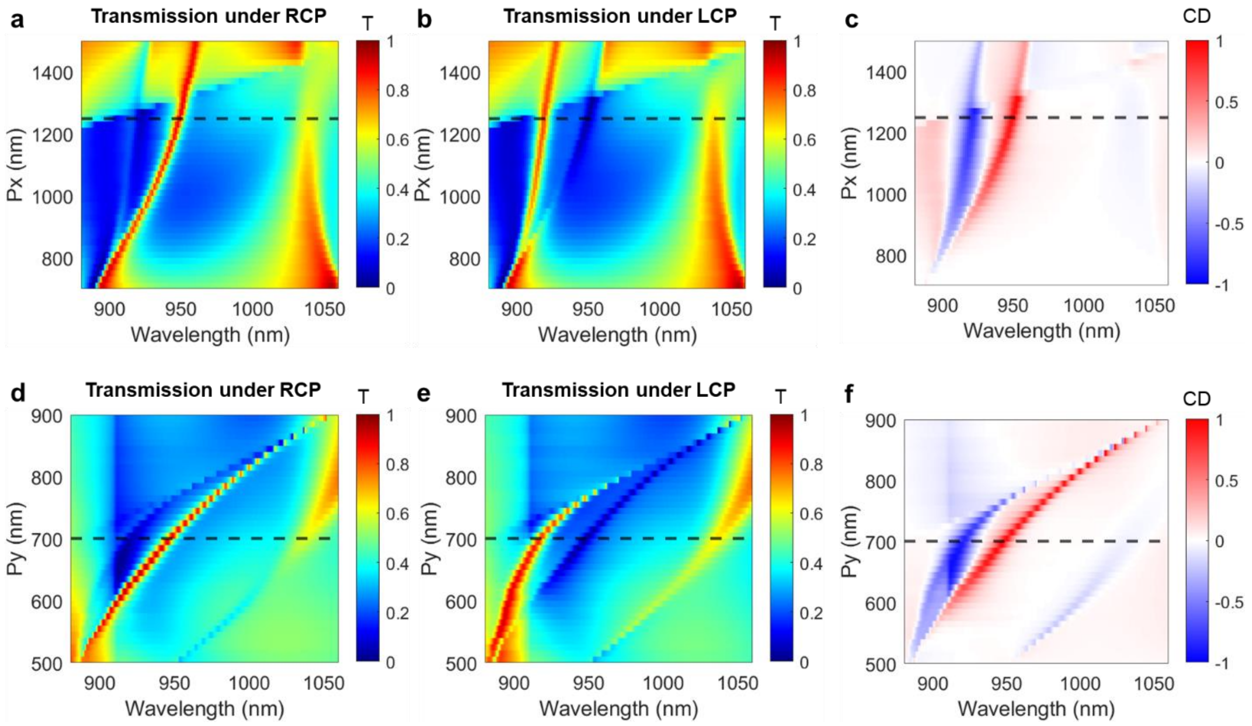

Figure S5. Transmission spectra for period in x and y direction. **a-c.** Transmission spectra under RCP (a), LCP (b), and CD (c) obtained by varying Px and keeping Py fixed to be 700 nm. The dashed line indicates the designed period of 1250 nm. **d-f.** Transmission spectra under RCP (d), LCP (e), and CD (f) obtained by varying Py and keeping Px fixed to be 1250 nm. The dashed line indicates the designed period of 700 nm.

Supplementary Note 6. Analysis of the nonlocal resonance

To confirm the effect of nonlocal resonance, the optical field intensities of resonance and their dependence on the incident angles are investigated (**Figure S6**). Metasurface with identical cross-sectional geometry with 100 nm height is modeled and analyzed. As described in **Supplementary Note 3**, **Figure 2g**, and **i**, lowering the height while keeping other parameters unchanged eliminates the influence of local mode and enables us to exclusively investigate the effect of nonlocal mode.

Figure S6a and **b** depict the transmission spectrum and field distribution at the resonant frequency of the metasurface. A relatively high-Q nonlocal mode is observed near 890 nm, and the excitation of a near-identical optical field distribution is exhibited under the incident polarization of both the x- and y-directions.

Because of the strong interaction between the adjusted meta-atoms by the nonlocal mode particularly in y-direction, the simulated metasurface becomes sensitive to the changes in incident angles due to the lateral k-vector by momentum generation. **Figure S6c** and **d** show the shifts of resonant wavelengths as the incident angle increases from zero to 20°. The nonlocal modes launched by both the x- and y-directed polarized light show the split and shift of resonance frequency by the changes in the incident angle.

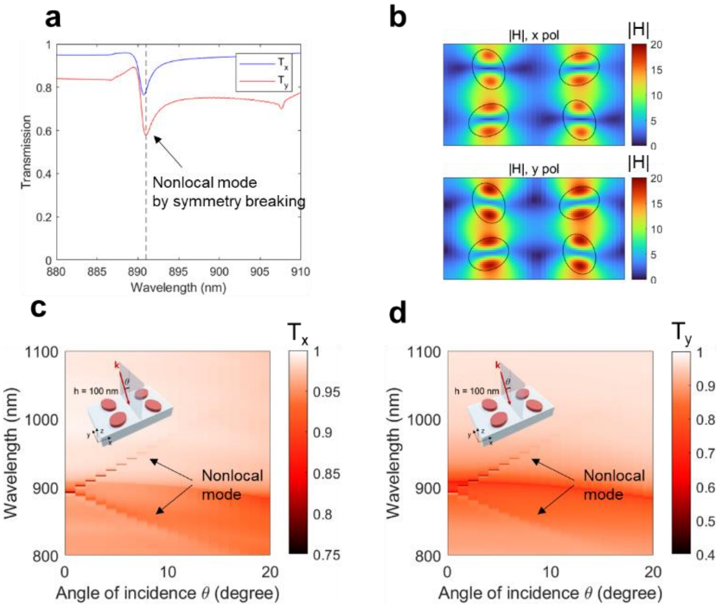

Figure S6. Analysis of the nonlocal resonance. **a.** Transmission spectra of the chiral metasurface with 100 nm height, which solely support nonlocal resonance. **b.** Simulated magnetic field distributions of the simulated nonlocal metasurface at the resonant wavelength of 891 nm. **c, d.** Simulated transmission spectra versus incident angles under x-polarized light (c) and y-polarized light (d).

Supplementary Note 7. Multipole decomposition and the formation of Fano resonance

To elucidate the formation process of local and nonlocal modes, we conduct multipolar decomposition of the proposed chiral metasurfaces. The decomposition analysis was performed based on the unit cell of nanoposts under periodic conditions.

First, in the non-perturbed state with an array of circular nanoposts, dipole modes by the local resonances are excited with the dominant contribution of electric dipole (ED) and magnetic dipole (MD) to the formation of broadband reflection spectrum (**Figure S7a** and **b**).

Secondly, as the degree of perturbation increases by the breaking of geometric symmetry, spectrally sharp nonlocal resonance arises. For example, the perturbation with δ of 5 nm induces high-Q spectral features due to ED modes near 920 nm, 950 nm, and an MD mode near 1050 nm (**Figure S7c** and **d**). The scattering power of such high-Q resonance is larger by one order of magnitude than the power of local resonances. The insets of **Figure S7c** and **d** which illustrate the magnified spectra show that such high-Q nonlocal peaks are overlaid on the background of local resonances, and such spectral coexistence of local and nonlocal modes facilitates the formation of Fano resonance. The designed metasurface is operated near the wavelength of 950 nm associated with the ED mode as indicated by the shaded region in the spectra. As the perturbation δ increases to 25 nm (**Figure S7e** and **f**), the magnitude of scattered power and Q-factor of the resonance decreases significantly, which results in relatively broader Fano spectra.

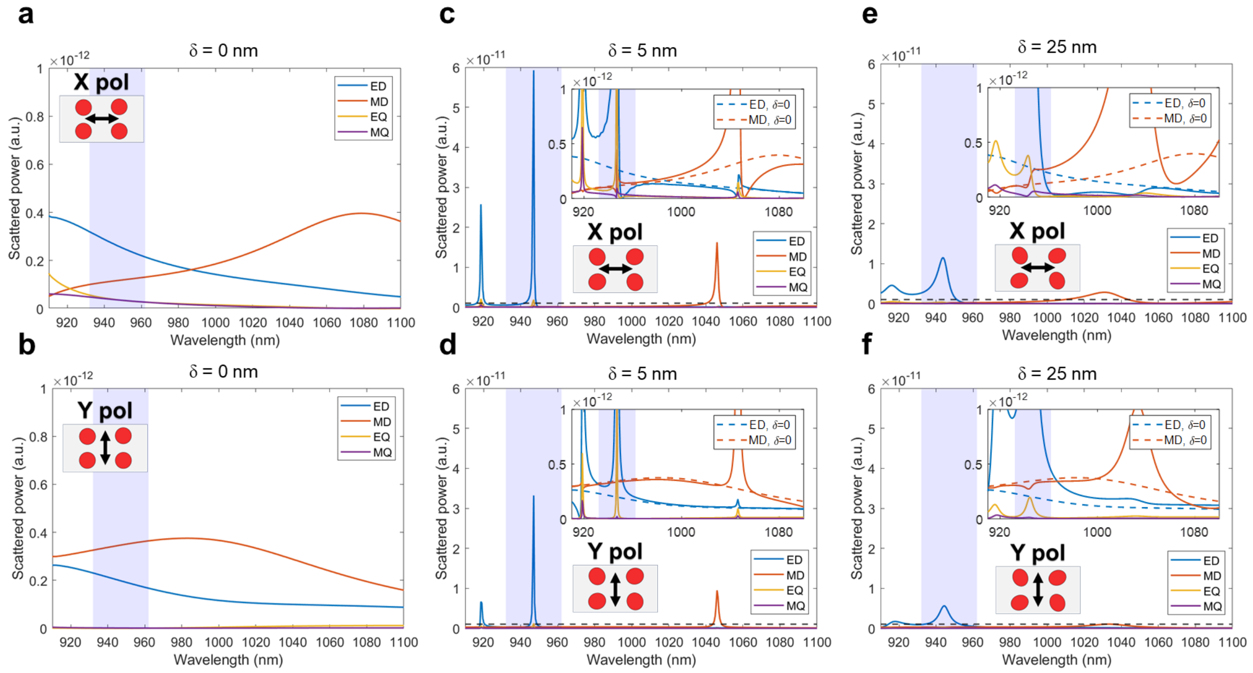

Figure S7. Multipole decompositions of the proposed chiral metasurfaces. **a-f.** Calculated scattering power under x-polarized (**a, c, e**) and y-polarized (**b, d, f**) light with varying δ. **a** and **b** show the scattering power for the non-perturbed metasurface, while panels **c-f** represent the perturbed metasurface with δ values of 5 nm (**c, d**) and 25 nm (**e, f**). The insets in the figures (**c-f**) represent the magnified spectra of scattering power at the scale of panels **a** and **b** (black dotted line in **c-f**). It is observed that the high-Q nonlocal mode is overlaid on the spectra of the local mode (dotted line, δ=0). The shaded region indicates the operational range of the chiral metasurface with a circular dichroism (CD) greater than 0.

Supplementary Note 8. Analysis of C_2 and mirror symmetry broken group cell

The symmetry breaking to excite nonlocal chiral resonant mode can also be achieved using a C_2 and mirror symmetry broken group cell. We demonstrate that the presented chiral metasurfaces can be implemented by C_2 broken group cell design as illustrated in **Figure S8**.

Figure S8a illustrates the arrangement of C_2 broken meta-units with a major axis of 260 nm and a minor axis of 200 nm. Period X (Px) and Y (Py) are 1100 nm and 600 nm, respectively and the height is 290 nm. By exciting the nonlocal mode in this manner, similar to the case of C_4 broken group cells, it is possible to induce optimized cross-polarization. To further analyze the nonlocal mode, the magnitudes and real parts of complex magnetic field profiles are illustrated in **Figure S8c**. It is worth noting that the field distribution of the C_2 broken group cell is similar to the C_4 broken group cell structure described in the main text. The nanostructure at the top and the bottom have opposite orientations of magnetic dipoles.

As illustrated in **Figure S8d**, the arrangement of two ellipsoids in a combination of 0° and 90° excites dark resonance and transmits x-polarized light in the C_2-based metasurface (**Figure S8d**). Similarly, C_2 metasurfaces with axis orientations of 45° and 135° selectively transmit y-directed linearly polarized light. The interaction of such resonances with localized Mie resonance yields asymmetric Fano-shaped transmission spectra (**Figure S8e**). Moreover, a C_2-based metasurface with ellipses oriented at 22.5° and 112.5° induces Fano resonance under both x- and y-polarized light (**Figure S8f**), resulting in the exhibition of circular dichroism. Furthermore, we show the variations of transmittance and Q-factor with respect to the degree of symmetry breaking δ, as shown in **Figure S8g-i**.

With regards to the nano-fabrications, the C_4 broken group cell shows an advantage of a more uniform electron dose to a unit cell for consistent lithography and the realization of full Stokes parameters detection.

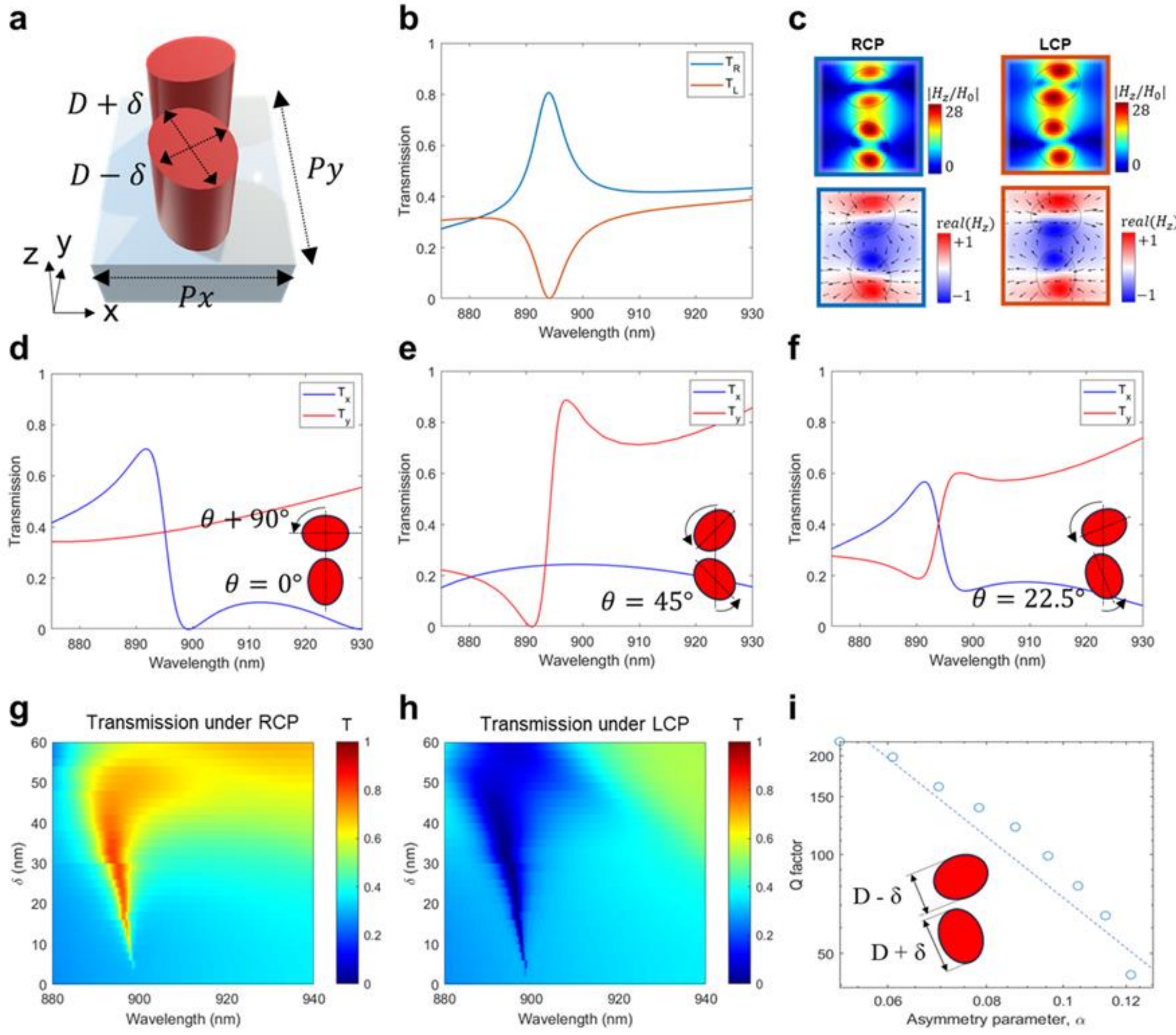

Figure S8. Analysis for a nonlocal metasurface with C_2 and mirror symmetry broken group cells. **a.** A schematic of a metasurface with C_2 symmetry breaking with the major axis being 260 nm and the minor axis being 200 nm. Period X (Px) and Y (Py) are 1100 nm and 600 nm, respectively, and height is 290 nm. **b.** Transmission spectra under the orthogonally polarized circular waves. **c.** Simulated magnetic field profiles under the incident light with RCP (left) and LCP (right) at the

operating wavelength of the maximum CD signal. **d-f.** Simulated transmission spectra displaying Fano resonance at targeted linear polarizations. The designed ellipses exhibit the oriented angles θ of 0°, 45°, and 22.5°, and adjacent ones are tilted by $\theta + 90°$. **g-h.** Transmission spectra under RCP (g) and LCP (h) incidence. **i.** Simulated Q-factors for the structures of different asymmetry parameters.

Supplementary Note 9. A combination of chiral metasurfaces for the consistent detection of S_3 without the variations of transmissions along the path of the equator of Poincaré spheres.

Simulated transmission spectra of two orthogonal chiral metasurfaces indicated by the dashed black line in **Figure S9a** are illustrated in **Figure S9b** and **c** for various SOP at the wavelength of 946 nm. To extract only the information of the degree of ellipticity angle regardless of the azimuthal angle, we average the transmission of the two composed metasurfaces as illustrated in **S9b**, **c,** and **d**. The combination of the two orthogonal forms of chiral metasurfaces eliminates the fluctuations of transmission along the azimuthal path near the equator to be zero (**Figure S9d**).

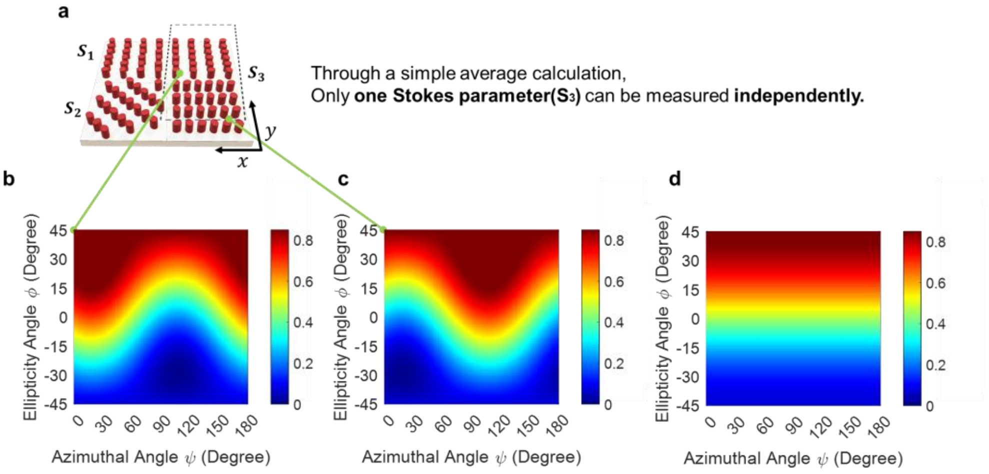

Figure S9. A system consisting of two orthogonal, chiral metasurfaces for the consistent detection of a Stokes parameter of circular polarization (S_3) without the variations of transmission along the path of the equator of Poincaré spheres. **a.** The set of metasurfaces for S_3 detection is indicated as the black dotted line. **b, c.** Simulated transmission profiles of the two orthogonally designed chiral metasurfaces **d.** Averaged transmission profile for S_3 detection.

Supplementary Note 10. Numerical analysis for direct Stokes parameters measurements

Since the proposed Stokes metasurfaces exhibit monotonic changes in transmission along the azimuthal and elliptical path, the numerical method to determine SOPs in **Figure 6** is relatively simple by monitoring intersecting points at the Poincaré sphere from the measured transmission at each metasurface.

The measured transmission of metasurface S_1 (MS1) constrained the Stokes vector on a circle parallel to the S_2 & S_3 plane on the Poincaré sphere. Similarly, the response of metasurface S_2 (MS2) and metasurface S_3 (MS3) constrained the Stokes vector on a circle of S_1 & S_3 plane and S_1 & S_2 plane, respectively. The intersecting point from the three circular lines enables the determination of SOP with a relatively lower error rate.

Irrespective of the degree of transmission contrast between two orthogonal polarizations, normalized transmission technically affords the complete detection of SOPs with the following calibration process. To experimentally realize this, we implement Stokes parameter detection using a total of four metasurfaces, as illustrated in **Figure 5**. The transmission of MS1 and MS2 is designed for linear polarization, and the transmission of MS3 is used to measure circular polarization.

$$S_1 = \frac{2T_1-(T_{MS1\_max}+T_{MS1\_min})}{T_{MS1\_max}+T_{MS1\_min}}$$

$$S_2 = \frac{2T_2-(T_{MS2\_max}+T_{MS2\_min})}{T_{MS2\_max}+T_{MS2\_min}} \qquad \text{(Eq. S2)}$$

$$S_3 = \frac{2T_3-(T_{MS3\_max}+T_{MS3\_min})}{T_{MS3\_max}+T_{MS3\_min}}$$

Coefficients $T_{MS\boldsymbol{n}\_min}$ and $T_{MS\boldsymbol{n}\_max}$ ($\boldsymbol{n}$ = 1, 2, 3) indicate the maximum and minimum transmissivity of each metasurface (MS1, MS2, and MS3) that can be obtained by pre-calibrations. T_1, T_2, and T_3 represent the transmissivity of each metasurface of MS1, MS2, and MS3, respectively, under the incident light of various polarizations. To attain transmission T_3, the averaged transmission from the two constituent chiral metasurfaces is calculated as explained in **Supplementary Note 9**.

Supplementary Note 11. Statistical analysis of the measured Stokes parameters

We conduct the measurements 4 times for each identical set of input polarization states and extract the mean values of Stokes parameters for reliability, which is expressed in the error bars in **Figure 6**. Additionally, we present two metrics to assess the accuracy and consistency of the metasurfaces in the measurements.

Firstly, to evaluate the polarization accuracy, the difference between the input polarization and the measured mean polarization states are evaluated using the following formulas.

$$\Delta S_1 = |S_{1,measured} - S_{1,input}|$$
$$\Delta S_2 = |S_{2,measured} - S_{2,input}| \quad \text{(Eq. S4)}$$
$$\Delta S_3 = |S_{3,measured} - S_{3,input}|$$

The differences between the measured mean values and the input polarization values are presented in **Figure S10**. On average, estimated errors across the entire measurement are approximately 0.047 for S_1, 0.049 for S_2, and 0.024 for S_3, which are relatively low and reliable in tracking the route along the Poincaré sphere.

Secondly, to assess the consistency of the measurements, the standard deviation from the data of four measurements is evaluated and depicted as error bars in **Figure 6** and **Figure S11**. On average, the standard deviation across the entire measurement is calculated as 0.149 for S_1, 0.156 for S_2, and 0.077 for S_3. The instability in these measurements may arise from the imperfect fabrication which degrades detection abilities related to the subtle geometric perturbation. Several improvements could mitigate such instability such as enhancing the transmission contrast between the two orthogonal polarizations by advanced fabrications and measurements which are less susceptible to noise.

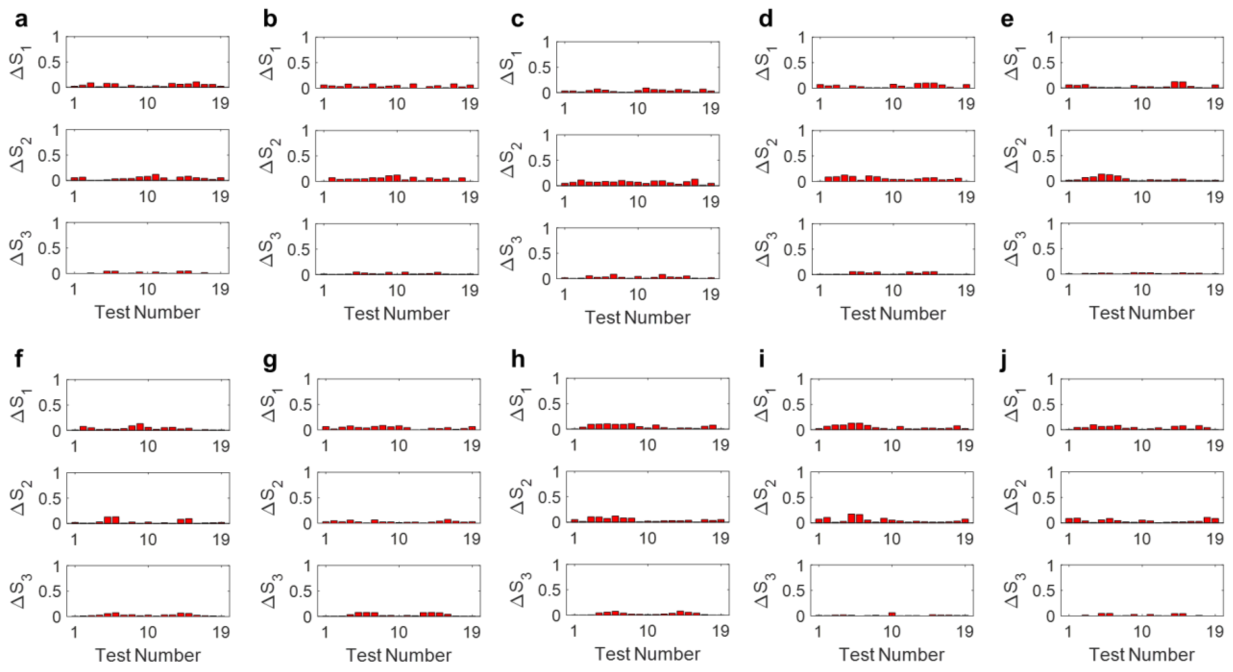

Figure S10. The differences between the measured mean values and the input polarization values for each SOPs. **a-j.** Results of the difference under various ellipticity angles on the Poincaré sphere with a 10-degree interval (Test Number 1 to 19). This is performed at 10 azimuthal angles with a 10-degree interval (from panel **a** to **j**), and the results correspond to the experimental data presented in **Figure 6**.

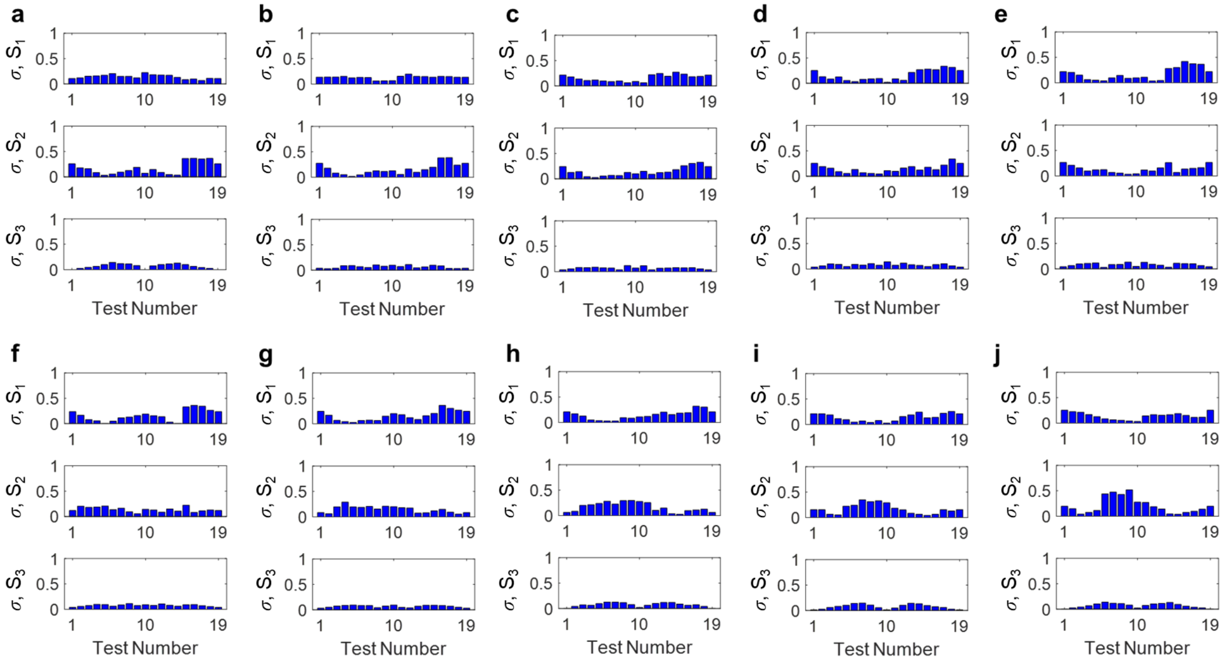

Figure S11. The standard deviation from the four-times measurements of each identical SOP**. a-j**. Standard deviations under various ellipticity angles on the Poincaré sphere with a 10-degree interval (Test Number 1 to 19). This is performed at 10 azimuthal angles with a 10-degree interval (from panel **a** to **j**), and the results correspond to the experimental data presented in **Figure 6**.

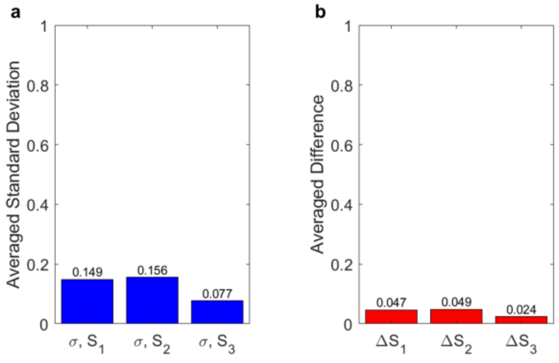

Figure S12. Averaged standard deviations (**a**) and averaged differences (**b**) of three Stokes parameters.

Supplementary Note 12. Impact of the fabrication imperfections

Due to the minimum geometric perturbation, the suggested chiral metasurface is more robust to fabrication imperfections such as the tilted etching of the nanostructures, compared to the metasurfaces with nanoscaled chiral geometries.

Figure S13 shows the transmission contrast and CD of metasurfaces with various slanted profiles of nanoposts by undercut or overcut effects. Although the resonant wavelength is red shifted caused by the expansion of volume by overcut effect, the magnitudes and linewidths of CD remain consistently preserved for the overall condition of vertical profiles.

Contrary to the robustness of etching profiles, the nature of nonlocal resonance strongly depends on the uniformity of nanostructure ensembles. Therefore, non-uniformities between constituent unit cells can lead to the degradation of nonlocal modes.[2] As exhibited in **Figure S14** and **S15**, the proposed metasurface shows degraded performance by the effect of non-uniformities.

To analyze the impact of such factors, we first investigate the effect of surface roughness by modeling the height variation between the constituent 64 nanoposts within the 16 unit cells as illustrated in **Figure S14**. FDTD simulations of the unit cells are performed under periodic boundary conditions. The heights of individual nanoposts are randomly assigned based on a normal distribution with a mean value of 330 nm and four different values of standard deviations. The distributed heights for each simulation are exhibited by the dashed line with the specific number of nanoposts as blue rectangular bars. As the irregularity increases, the transmission contrast between RCP and LCP decreases with the broadening of the linewidth of resonances. The simulated analysis expects a decrease in transmission contrast from approximately 0.9 to 0.3 when the standard deviation exceeds 30 nm.

The impact of cross-sectional diameter variation is also evaluated with a similar method of approach for random size distributions to 64 nanopost elements as illustrated in **Figure S15**. A significant decrease in performance is observed as the standard deviation reaches 6 nm, and at the standard deviation of 9 nm, the transmission contrast decreases to 0.4. Using higher-quality films such as crystalline silicon film on oxides and optimization of uniform etching can mitigate such irregularities during the fabrication process.

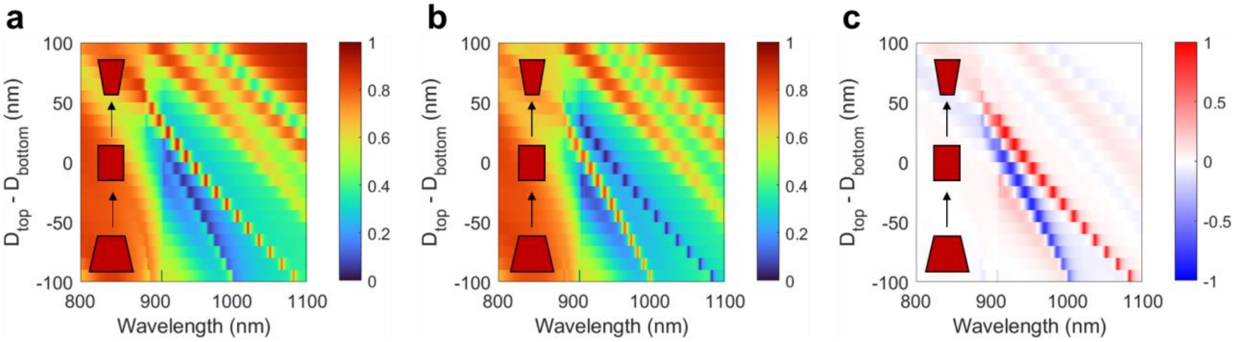

Figure S13. Analysis of the impact of undercut and overcut on the performance of the chiral metasurface. **a-c.** Transmission spectra under RCP (a), LCP (b), and circular dichroism (CD) of the chiral metasurfaces with overcut or undercut nanoposts. The top diameter (D_{top} = 240 nm) of the nanoposts was kept constant while varying the bottom diameter.

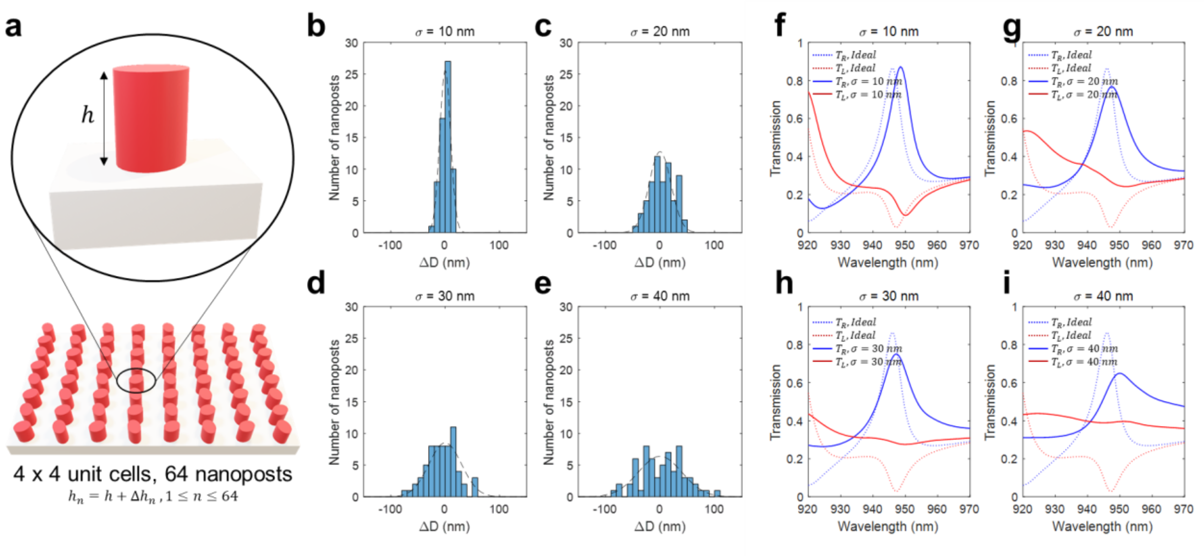

Figure S14. Analysis of performance degradation by the non-uniform height distribution during the fabrication process. **a.** Schematic of the simulated group cells. **b-e.** The height of each nanoposts is set differently. Height distributions of the simulated group cell. **f-i.** Simulated transmission under RCP and LCP for four group cells with different height distributions. The dotted line represents the ideal case.

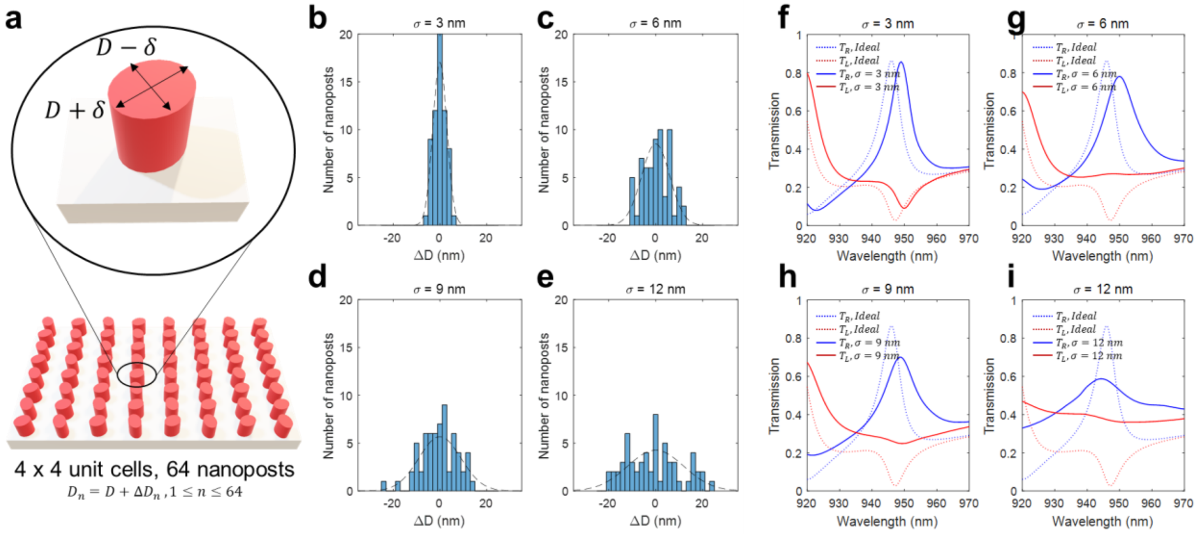

Figure S15. Analysis of performance degradation by the diameter variations. Schematic of the simulated group cell. **a.** The diameter (D) of each nanoposts is set differently for the performance evaluation. **b-e.** Diameter distributions of the simulated group cell. **f-i.** Simulated transmission under RCP and LCP with four different diameter distributions. The dotted line represents the ideal case of a uniform size of diameter.

Supplementary Note 13. Fabrication steps

We fabricated the array operating at the near-infrared wavelength. The 330 nm thick poly-Si was deposited on the quartz substrate followed by EBL to define the elliptical shape. Next, a 30 nm thick Cr hard mask was deposited, followed by a lift-off process. The remaining elliptical Cr pattern is used as a mask. Finally, we etched Poly-Si with an ICP-RIE machine and removed the remaining Cr pattern by wet-etching. The design of planar and near-circular arrays affords monolithic fabrication with consistent-sized nanolithography for all samples.

As an alternative fabrication step to potentially achieve a higher level of sample quality, chromium (Cr) film can be pre-deposited before e-beam lithography. This increases the conductivity of the film and alleviates the e-beam charging effects to realize a more uniformly defined size of nanostructures. Using the e-beam resist as a mask, pre-deposited Cr film can be etched to serve as a hard mask for silicon etching.

In addition, the use of a crystalline silicon film, such as a SOS (Silicon on Sapphire) by wafer bonding process, can significantly improve the quality of the fabricated samples.

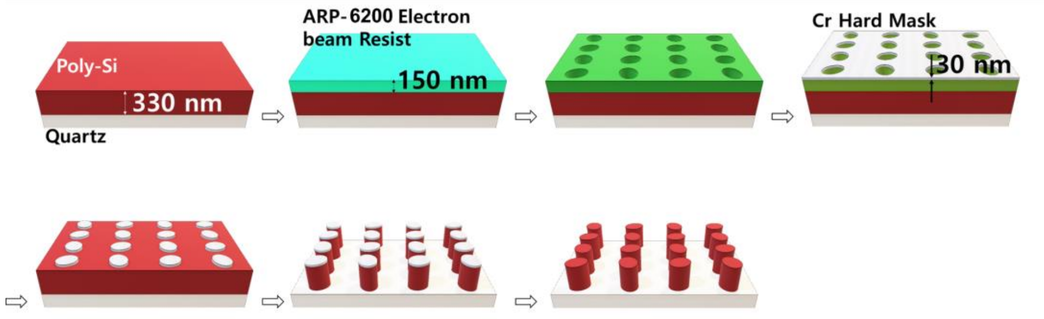

Figure S16. Fabrication steps of the array of nanopost.

Supplementary Note 14. The impact of the unit cell numbers on the collective oscillations

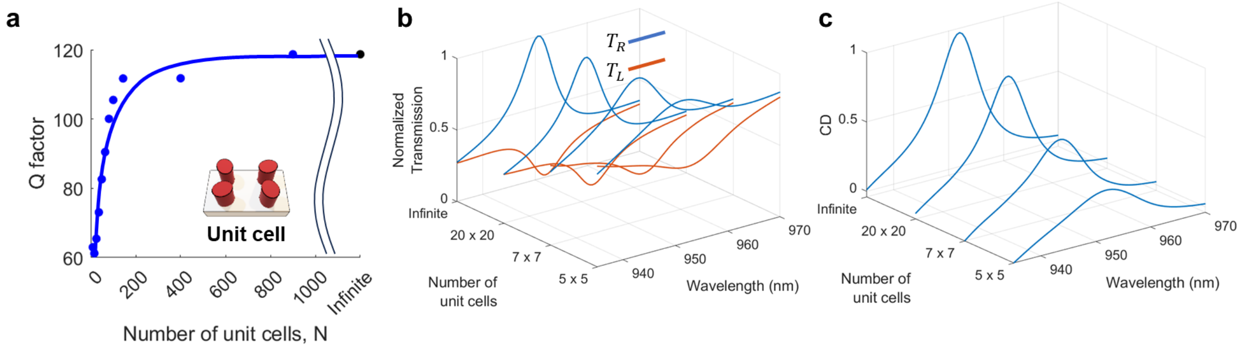

Figure S17. The impact of the number of unit cells on the performance of the metasurface. **a.** Evaluations of the Q factors versus the number of unit cells. The increase in the number of unit cells enhances the Q factor due to the interaction between adjacent nanoposts and collective oscillations of resonant dipoles. **b**. Normalized transmission under RCP (blue line) and LCP (orange line) for different numbers of unit cells. **c.** CD spectra for different numbers of unit cells.

Supplementary note 15. Effect of overcut shape with various degrees of perturbations

To find the physical origin of the opposite trends of wavelength shift between simulation and experiment in **Figure 4c** and **4d**, various nanoposts with an ideal shape and overcut shapes are modeled with the analysis of their effects on the spectral shift. In the ideal structure, an increase in the perturbation δ leads to a blue shift of the resonant peak due to the slightly reduced cross-sectional area of the ellipse. In the case of the overcut structure, the trend of red shift can be observed opposite to the trend of ideal structures.

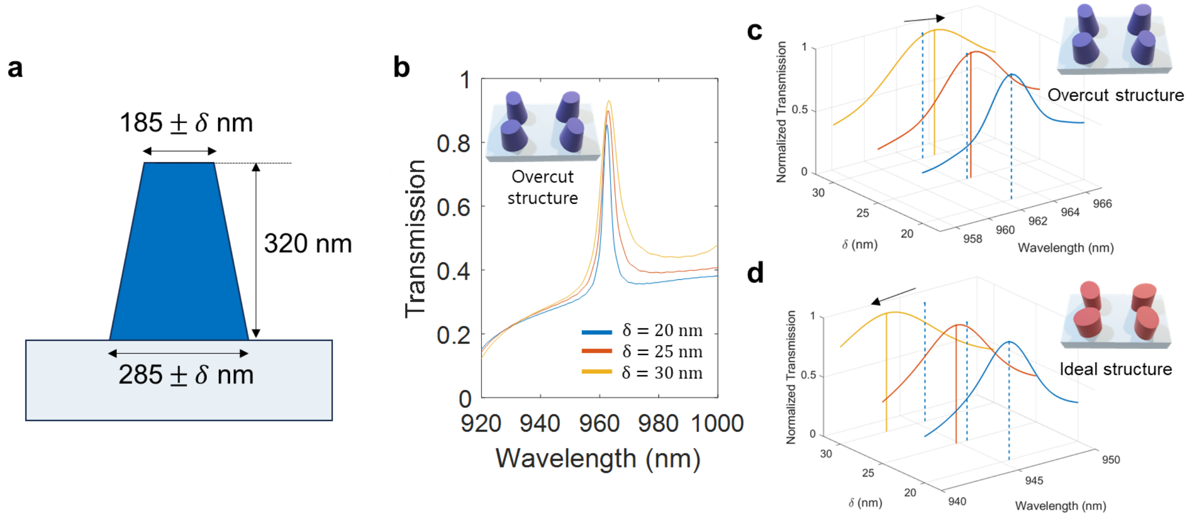

Figure S18. Difference in the wavelength shift direction due to the changes in the perturbation δ. **a.** Simulated vertical cross-section of an overcut nanopost. The bottom diameter of the nanopost is 100 nm larger than the top diameter. **b.** The change in transmission spectra under RCP light with varying δ values. **c, d.** Comparison of the shift trends with perturbation variations for the overcut structure (c) and the ideal structure (d). The dotted vertical line represents the resonant frequency at δ to be 20.

Supplementary Note 16. Experiments for Jones matrix transmission spectra

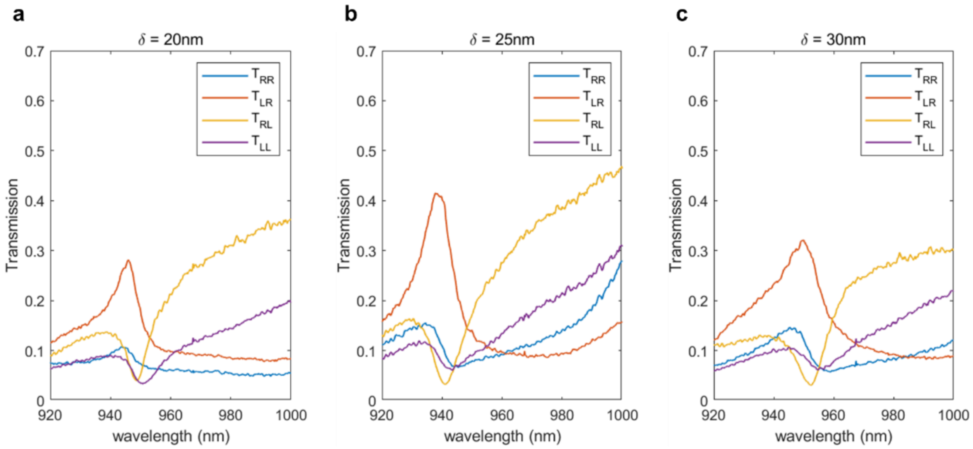

Figure S19. Experimental results showing the transmission spectra for different components of polarizations under the incident light with circular polarization. **a, b, c.** Transmission spectra of metasurfaces with δ = 20 nm (**a**) δ = 25 nm (**b**) δ = 30 nm (**c**). The experimental results approximately match the simulated analysis in **Figure 3i** in the main text. In all three cases, the T_{LR} component exhibits the highest transmission, while the other components are suppressed.

Supplementary Note 17. Experimental setup

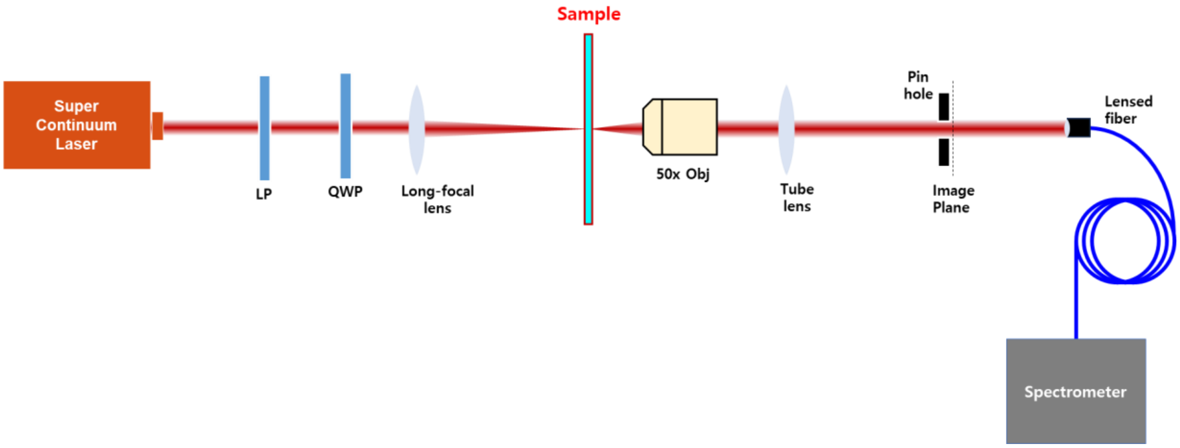

Figure S20. Experimental setup for the nonlocal chiral and Stokes metasurfaces. LP stands for linear polarizer and QWP stands for quarter wave plate. Obj for the objective lens. The image plane with a pinhole is positioned apart from the tube lens by focal length.

Supplementary Note 18. Dependence on the incident angle of metasurfaces

Figure S21 illustrates the dependence on the incident angle of chiral metasurfaces. The heights of the nanopost array are modeled as 160 nm, 220 nm, and 330 nm. As illustrated in the insets of each panel, induced optical field distributions are similar to each other under incident RCP, which implies that the similar nonlocal resonances are excited by symmetry breaking.

For the height of 160 nm (**Figure S21c** and **d**), the resonant mode is weakened as the incident angle is tilted from 0 to 3° in the x-direction (**Figure S21c**). In **Figure S21d**, the resonant dip is red-shifted by the excitation of the y-directed k vector due to the lateral momentum generation. Such angle-sensitive properties of metasurfaces are additionally analyzed and illustrated in **Supplementary Note 6**. Contrary to the angle sensitivity of the previous analysis, as the height of the metasurface increases up to 330 nm, the resonance is not significantly altered by the changes in incident angles (**Figure S21g** and **h**).

Figure S22 further shows the degree of robustness to the incident angle for the metasurface with h = 330 nm. Within the incident angle of less than 5°, the strength and wavelength of resonance remain unchanged along with the consistent CD spectra.

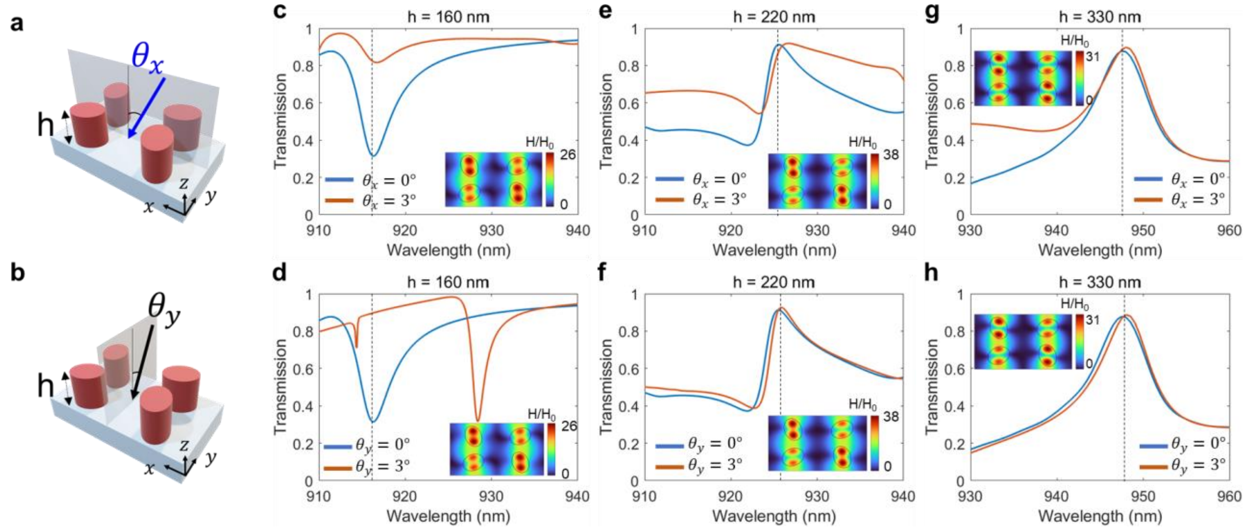

Figure S21. Effect of incident angles on the optical properties of chiral metasurfaces. **a, b.** Schematics of the proposed chiral metasurface with the oblique incident light towards x- and y-directions. **c-h.** Transmittance of chiral metasurfaces with various heights under RCP illumination. The insets within each figure illustrate magnetic field distributions under normally incident light at the resonant frequency. Nanopost heights are modeled as h = 160 nm (c, d), h = 220 nm (e, f), and h = 330 nm (g, h).

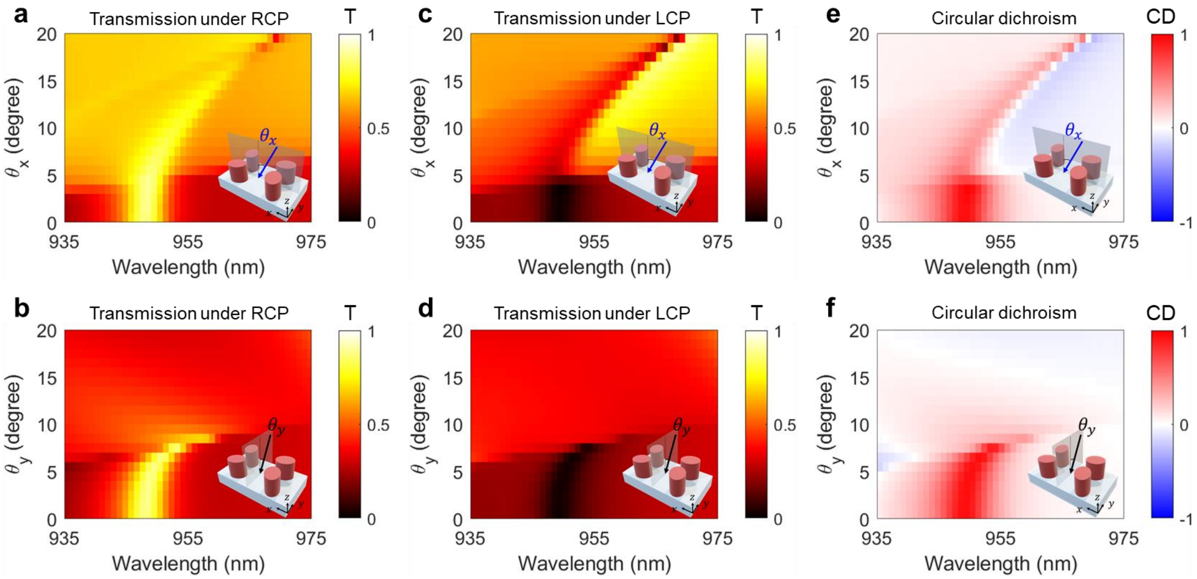

Figure S22. Simulated transmission of the chiral metasurface with h = 330 nm under various incident angles. **a-d.** Transmission under RCP (a, b) and LCP (c, d) light. **e, f.** Circular dichroism (CD) for incident light tilted in the x-direction (e) and y-direction (f).

Supplementary Note 19. Comparison with previously reported chiral metasurfaces.

Table S1. Chiral metasurfaces in transmission mode.

**Ref.**	**Geometric shapes of the meta-atoms**	**Planar design**	**Operating wavelength**	**Materials**	**CD ($\frac{T_R-T_L}{T_R+T_L}$) (Sim/Exp)**	**FWHM (Sim/Exp)**	**Stokes detection**
4	Multilayered elliptical hole	X	744, 896 nm	Ag	*~0.52/0.58**	~ 80 nm / ~ 82 nm	X
5	Gammadion nanostructure	O	~ 540 nm	TiO_2	*0.90/0.80**	~ 18 nm / ~ 16 nm	X
6	Slant etched C-shaped hole	X	~ 760 nm	Au	0.85/0.78	~ 67 nm / ~ 83 nm	X
7	Z-shaped nanostructure	O	1600 - 1700 nm	Ge	0.95/0.80	~ 35 nm / NA	X
8	U-shaped folded nanostructure	X	~ 5760 nm	Si_3N_4, Au	*0.77/0.65**	~ 500 nm / ~ 500 nm	X
9	3D Archimedean spiral	X	2000-5000 nm	Si_3N_4, Au	0.39/0.35	NA / ~500 nm	X
10	Vertically combined nanograting and nanobrick array	X	1400 - 1600 nm	Au, Si	~0.99 / ~0.93	~ 300 nm / ~ 300 nm	O
11	Multilayered nanobricks	X	1420, 1500 nm	Si	~0.71 / ~0.80	~ 110 nm / ~ 113 nm	X
12	Two differently oriented nanobricks	O	600-700 nm	Si	~0.99 / ~0.93	~ 40 nm / ~ 80 nm	O
13	Eight dimers with vertical offsets	X	596 nm	Si	1 / NA	~ 1 nm / NA	X
14	Membrane with circular and elliptical nanoholes	O	~ 870 nm	Si	~0.94 / ~0.23	~ 6 nm / ~ 5.3 nm	X
15	Multilayered elliptical hole	X	NA	Si	1 / NA	NA	X
16	Notched nanocylinder	O	~1380 nm	Si	0.98 / 0.88	~ 1 nm / ~ 2.83 nm	X
17	Two nanobricks with different height	X	~ 892 nm	Si	~0.99 / ~0.67	~ 7.5 nm / ~ 9.35 nm	X
**Our work**	**Near-circular ellipses with minimized perturbations**	**O**	**~ 950 nm**	**Si**	0.93 / 0.77	**6.7 nm / 12.2 nm**	**O**

$* \, CD = T_R - T_L$

Table S1 compares our proposed metasurface with previously reported chiral metasurfaces. It includes studies of both the local and nonlocal chiral metasurfaces, with nonlocal metasurfaces highlighted in blue shading. Recent studies employ chiral meta-atoms with a variety of configurations, such as slant etched holes, nanobricks with varying heights, notched designs, and multi-layered systems which impose a challenge of fabricating chiral nanostructures with consistent size and full Stokes parameters detection with a relatively high Q-factor.

For a more comprehensive comparison, **Table S2** shows more details of previously reported nonlocal chiral metasurfaces including chiral BIC with their unique approaches of inducing

symmetry breaking. Our proposed nonlocal chiral metasurface achieves comparable circular dichroism (CD) and relatively high-Q resonances by using a single-layered metasurface made of near-circular ellipses with relevant local resonances. Notably, our work demonstrates the first nonlocal metasurfaces based Stokes parameters detection at a targeted wavelength with high-Q spectra by minimally perturbing the geometry of meta-atoms with constant filling ratio and fabricating consistent-sized Stokes metasurfaces with identical electron dose for lithography.

Table S2. Nonlocal Chiral metasurfaces in transmission mode.

Ref.	Schematics of unit cells*	Symmetry breaking to induce high-Q chiral response	Operating wavelength	FWHM (Sim/Exp)	Stokes detection	Further applications
13	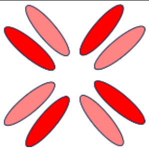	Tilted dimers with different heights inducing out-of-plane symmetry breaking	596 nm	~ 1 nm / NA	X	NA
14	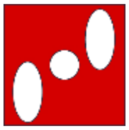	Photonic crystal membrane with elliptical and circular nanoholes	~ 870 nm	~ 6 nm / ~ 5.3 nm	X	Circular polarization-resolved imaging
15	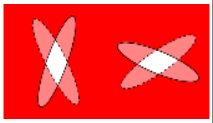	Two-layered membrane with twisted positions of elliptical nanoholes	NA	NA	X	Beam shaping with geometric phase
16	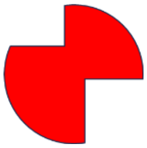	Nanopost with two notches which have slightly different sizes of lengths	~1380 nm	~ 1 nm / ~ 2.83 nm	X	Nonlinear circular dichroism
17	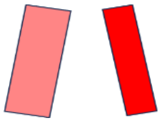	Tilted two nanobricks with different heights inducing out-of-plane symmetry breaking	~ 892 nm	~ 7.5 nm / ~ 9.35 nm	X	NA
**Our work**	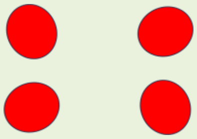	**Identical elliptical nanoposts with minimal perturbations**	**~ 950 nm**	**6.7 nm / 12.2 nm**	O	**Direct full Stokes parameters detections**

*Different colors in schematics represent the difference in height(out-of-plane) of the unit cells

Supplementary Note 20. Nonlocal chiral metasurfaces for oblique excitation.

Nonlocal mode by oblique excitation can make chirality in transmission as shown in **Figure S23**. The metasurface is composed of silicon elliptical nanoposts with a major axis of 280 nm, a minor axis of 200 nm, and a height of 350 nm. The elliptical nanoposts are arranged in a rectangular lattice with Px of 625 nm and Py of 350 nm to make birefringence as analyzed in **Supplementary Note 1**. Then, the tilted angle of each meta-atom is set to 45° to induce cross-polarization for chiral response. As a result, Once the BIC mode shown in **Figure S23b** is excited by oblique incident light, High-Q circular dichroism in the transmission is made by the same effect as the structure in the main text (**Figure S23a**). The colormap in **Figures S23c** and **d** illustrates the variations in transmission spectra for the changes in the incident angle of light. It shows a gradual increase of FWHM in transmissions as θ_y increases as in the analysis in **Supplementary Note 4**.

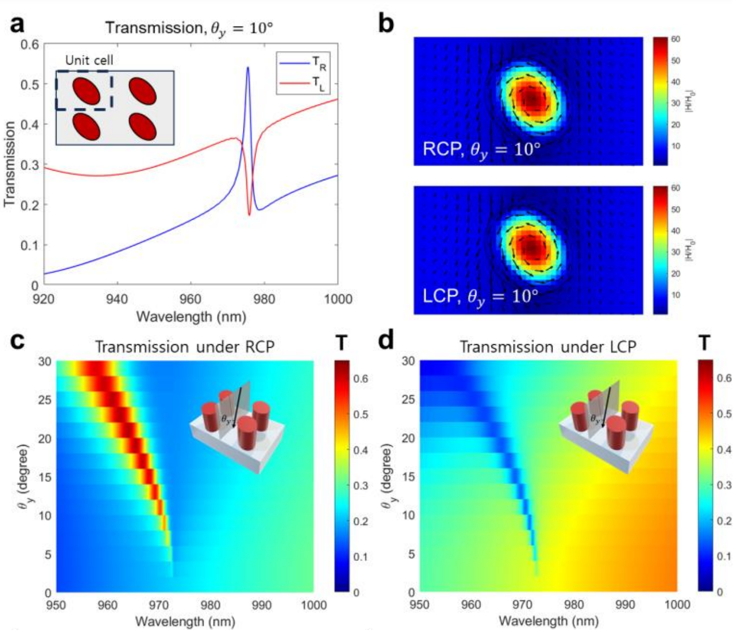

Figure S23. Nonlocal chiral response without symmetry breaking under oblique incident light. **a.** Simulated transmission spectra of the metasurface without symmetry breaking. The inset shows the schematic of the unit cell. **b.** Magnetic field distributions of metasurface under circularly

polarized oblique excitation. The black arrows indicate in-plane electric field components. **c, d.** Simulated transmission of the metasurface without symmetry breaking under various incident angles under RCP (c) and LCP (d) light.

Supplementary note 21. Dependence of circular dichroism on extinction coefficient by absorption

To analyze the dependence of circular dichroism on the material's extinction coefficient, we varied the material's extinction coefficient while keeping the index value constant at 3.59. As the extinction coefficient increased from 0.001 (blue line) to 0.02 (purple line), transmission under RCP gradually decreased. However, transmission under LCP exhibited a minimum value at an extinction coefficient of 0.01 (yellow line) because it is the nearest value of critical coupling. Also, circular dichroism in **Figure S24b** shows the maximum value at the same extinction coefficient.

To better illustrate the trend, we depicted **Figure S24c** to show circular dichroism and the minimum transmission value under LCP changes with the extinction coefficient. We observed that as the extinction coefficient approaches the critical coupling condition, CD increases, and as it moves away, CD decreases.

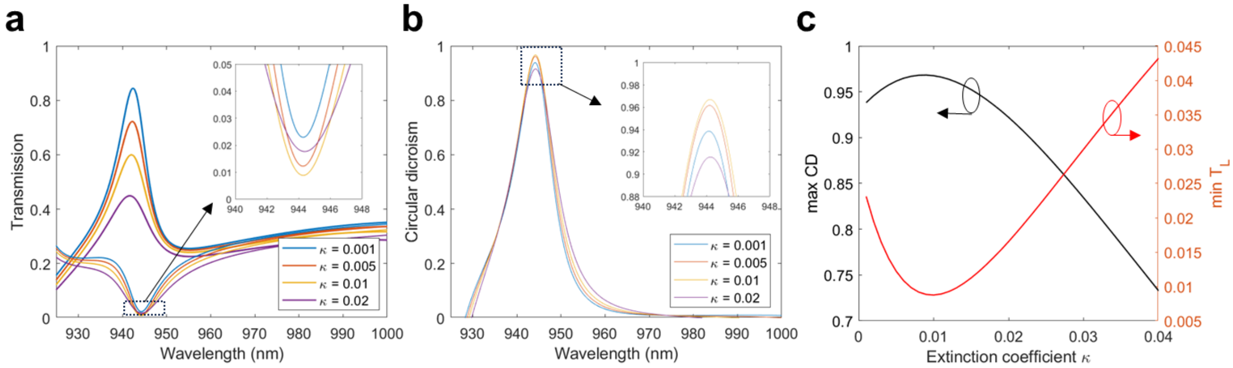

Figure S24. Analysis of the impact of change in material absorption rate in circular dichroism. **a.** Simulated transmission spectra under RCP and LCP in various conditions of extinction coefficient. **b.** Simulated circular dichroism in various extinction coefficients of silicon. **c.** Dependence of maximum circular dichroism and minimum transmission under LCP on extinction coefficients by absorption ratio.

Supplementary References

1. Koshelev, K.; Lepeshov, S.; Liu, M.; Bogdanov, A.; Kivshar, Y. Asymmetric metasurfaces with high-Q resonances governed by bound states in the continuum. *Phys. Rev. Lett.* **2018**, *121* (19), 193903.

2. Kühne, J.; Wang, J.; Weber, T.; Kühner, L.; Maier, S. A.; Tittl, A. Fabrication robustness in BIC metasurfaces. *Nanophotonics* **2021**, *10* (17), 4305-4312.

3. Malek, S. C.; Overvig, A. C.; Alù, A.; Yu, N. Multifunctional resonant wavefront-shaping meta-optics based on multilayer and multi-perturbation nonlocal metasurfaces. *Light: Sci. Appl.* **2022**, *11* (1), 246.

4. Rodrigues, S. P.; Lan, S.; Kang, L.; Cui, Y.; Panuski, P. W.; Wang, S.; Urbas, A. M.; Cai, W. Intensity-dependent Modulation of Optically Active Signals in a Chiral Metamaterial. *Nat. Commun.* **2017**, 8 (1), 14602.

5. Zhu, A. Y.; Chen, W. T.; Zaidi, A.; Huang, Y.-W.; Khorasaninejad, M.; Sanjeev, V.; Qiu, C.-W.; Capasso, F. Giant Intrinsic Chiro-optical Activity in Planar Dielectric Nanostructures. *Light: Sci. Appl.* **2018**, 7 (2), 17158-17158.

6. Chen, Y.; Gao, J.; Yang, X. Chiral Metamaterials of Plasmonic slanted nanoapertures with symmetry breaking. *Nano Lett.* **2018**, 18 (1), 520-527

7. Ma, Z.; Li, Y.; Li, Y.; Gong, Y.; Maier, S. A.; Hong, M. All-dielectric Planar Chiral Metasurface with Gradient Geometric Phase. *Opt. Express* **2018**, 26 (5), 6067-6078.

8. Yang, S.; Liu, Z.; Hu, S.; Jin, A.-Z.; Yang, H.; Zhang, S.; Li, J.; Gu, C. Spin-selective Transmission in Chiral Folded Metasurfaces. *Nano Lett.* **2019**, 19 (6), 3432-3439.

9. Tseng, M. L.; Lin, Z. H.; Kuo, H. Y.; Huang, T. T.; Huang, Y. T.; Chung, T. L.; Chu, C. H.; Huang, J. S.; Tsai, D. P. Stress-induced 3D chiral fractal metasurface for enhanced and stabilized broadband near-field optical chirality. *Adv. Opt. Mater.* **2019**, *7* (15), 1900617.

10. Basiri, A.; Chen, X.; Bai, J.; Amrollahi, P.; Carpenter, J.; Holman, Z.; Wang, C.; Yao, Y. Nature-inspired Chiral Metasurfaces for Circular Polarization Detection and Full-Stokes Polarimetric Measurements. *Light: Sci. Appl.* **2019**, 8 (1), 78.

11. Tanaka, K.; Arslan, D.; Fasold, S.; Steinert, M.; Sautter, J. r.; Falkner, M.; Pertsch, T.; Decker, M.; Staude, I. Chiral Bilayer All-dielectric Metasurfaces. *ACS nano* **2020**, 14 (11), 15926-15935.

12. Wang, S.; Deng, Z.-L.; Wang, Y.; Zhou, Q.; Wang, X.; Cao, Y.; Guan, B.-O.; Xiao, S.; Li, X. Arbitrary Polarization Conversion Dichroism Metasurfaces for All-in-one Full Poincaré Sphere Polarizers. *Light: Sci. Appl.* **2021**, 10 (1), 24.

13. Gorkunov, M. V.; Antonov, A. A.; Kivshar, Y. S. Metasurfaces with maximum chirality empowered by bound states in the continuum. *Phys. Rev. Lett.* **2020**, *125* (9), 093903.

14. Semnani, B.; Flannery, J.; Al Maruf, R.; Bajcsy, M. Spin-preserving chiral photonic crystal mirror. *Light: Sci. Appl.* **2020**, *9* (1), 23.

15. Overvig, A.; Yu, N.; Alù, A. Chiral Quasi-bound States in the Continuum. *Phys. Rev. Lett.* **2021**, 126 (7), 073001.

16. Shi, T.; Deng, Z.-L.; Geng, G.; Zeng, X.; Zeng, Y.; Hu, G.; Overvig, A.; Li, J.; Qiu, C.-W.; Alù, A.; Kivshar, Y. S.; Li, X. Planar Chiral Metasurfaces with Maximal and Tunable Chiroptical Response Driven by Bound States in the Continuum. *Nat. Commun.* **2022**, 13 (1), 4111.

17. Kühner, L.; Wendisch, F. J.; Antonov, A. A.; Bürger, J.; Hüttenhofer, L.; de S. Menezes, L.; Maier, S. A.; Gorkunov, M. V.; Kivshar, Y.; Tittl, A. Unlocking the out-of-plane dimension for photonic bound states in the continuum to achieve maximum optical chirality. *Light: Sci. Appl.* **2023**, *12* (1), 250.
